# Dithiolene Complexes of First‐Row Transition Metals for Symmetric Nonaqueous Redox Flow Batteries

**DOI:** 10.1002/cssc.201901702

**Published:** 2019-09-03

**Authors:** Ross W. Hogue, Craig G. Armstrong, Kathryn E. Toghill

**Affiliations:** ^1^ Department of Chemistry Lancaster University Lancaster LA1 4YB United Kingdom

**Keywords:** batteries, dithiolenes, electrochemistry, nonaqueous systems, redox chemistry

## Abstract

Five metal complexes of the dithiolene ligand maleonitriledithiolate (mnt^2−^) with M=V, Fe, Co, Ni, Cu were studied as redox‐active materials for nonaqueous redox flow batteries (RFBs). All five complexes exhibit at least two redox processes, making them applicable to symmetric RFBs as single‐species electrolytes, that is, as both negolyte and posolyte. Charge–discharge cycling in a small‐scale RFB gave modest performances for [(tea)_2_V_mnt_], [(tea)_2_Co_mnt_], and [(tea)_2_Cu_mnt_] whereas [(tea)Fe_mnt_] and [(tea)_2_Ni_mnt_] (tea=tetraethylammonium) failed to hold any significant capacity, indicating poor stability. Independent negolyte‐ and posolyte‐only battery cycling of a single redox couple, as well as UV/Vis spectroscopy, showed that for [(tea)_2_V_mnt_] the negolyte is stable whereas the posolyte is unstable over multiple charge–discharge cycles; for [(tea)_2_Co_mnt_], [(tea)_2_Ni_mnt_], and [(tea)_2_Cu_mnt_], the negolyte suffers rapid capacity fading although the posolyte is more robust. Identifying a means to stabilize V_mnt_^3−/2−^ as a negolyte, and Co_mnt_^2−/1−^, Ni_mnt_^2−/1−^, and Cu_mnt_^2−/1−^ as posolytes could lead to their use in asymmetric RFBs.

## Graphical abstract

**Both positive and negative**: Symmetric nonaqueous redox flow batteries employing metal–dithiolene complexes as the redox‐active species in both the negative and positive half‐cells are demonstrated with five metal complexes of the dithiolene ligand maleonitriledithiolate (mnt^2−^) with M=V, Fe, Co, Ni, Cu. All five complexes exhibit at least two redox processes, making them applicable to symmetric RFBs as single‐species electrolytes, that is, as both negolyte and posolyte.

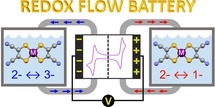

## Introduction

To meet rising global energy demands and to reduce fossil fuel consumption, renewables such as solar and wind are being increasingly implemented as energy sources. However, owing to their intermittent nature, efficient and cost‐effective grid‐scale energy storage is required before solar and wind energy can achieve widespread implementation.^1^, ^2^ A promising candidate for grid‐scale energy storage is the redox flow battery (RFB) technology, whereby solutions of electroactive materials are pumped to/from external tanks to the electrode interface for charging/discharging.^3^, ^4^, ^5^ As energy is stored externally to the electrochemical reactor, the capacity can be increased independently of the battery power. At present, commercial RFBs utilize aqueous electrolyte solutions of inorganic metal salts, however, despite continual progress in power outputs and efficiencies being made, the cell potential is inherently limited by the narrow (1.23 V) electrochemical window of water. Instead, the development of nonaqueous RFBs, which use organic solvents with wide electrochemical windows, is anticipated to improve the voltage outputs.^6^, ^7^, ^8^, ^9^, ^10^ Acetonitrile (MeCN) is an attractive solvent for nonaqueous RFBs, and is the main solvent of choice here, owing to its wide (≈5 V) electrochemical window as well as low viscosity (0.34 vs. 0.89 MPa s for water) and moderate dielectric constant (35.9 vs. 78.4 for water).^11^

Metal–ligand coordination complexes are good candidates for nonaqueous RFB electrolytes as they can be stable in multiple oxidation states and have high solubility in organic solvents. Furthermore, careful choice of metal ion as well as modification of the ligand scaffold (e.g., solubilizing groups, denticity, donor groups) can allow for fine tuning of the desired properties for RFB applications.^12^, ^13^, ^14^, ^15^ Indeed, several metal coordination complexes have been tested as electrolytes for nonaqueous RFBs with cell potentials in excess of 1.23 V; these contain acetylacetonate,^12^, ^16^, ^17^, ^18^, ^19^, ^20^, ^21^ bipyridine,^13^, ^15^, ^22^, ^23^, ^24^, ^25^ phenanthroline,^26^, ^27^ terpyridine‐like,^14^, ^15^ trimetaphosphate,^28^ and macrocyclic^29^, ^30^ ligands.

An attractive and simple RFB system, which avoids cross‐contamination of the two electrolyte solutions through membrane crossover, employs a single species electrolyte in a symmetric cell—that is, a battery that uses only one species as both negolyte (electrolyte that is reduced on battery charging, that is, anolyte) and posolyte (oxidized electrolyte, that is, catholyte). In this approach, the battery does not suffer from irreversible capacity loss and self‐discharge through the mixing of electrolytes, and instead a rebalancing procedure to restore the original negolyte/posolyte composition can be performed, as is done in aqueous all‐vanadium RFBs.^31^ For this, the redox‐active species needs to have (at least) two redox processes and be stable across the three associated redox states. Dithiolene ligands are of particular interest here, as they are non‐innocent when bound to transition metal ions leading to complexes with multiple redox events, through oxidation and reduction centered on either the metal or dithiolene ligand.^32^, ^33^, ^34^, ^35^

**Figure 1:**
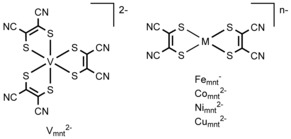
The chemical structures of metal–dithiolene complexes studied as single‐species electrolytes for nonaqueous RFBs in this work.

Recently, the vanadium complex of the dithiolene ligand 1,2‐dicyanoethylene‐1,2‐dithiolate (maleonitriledithiolate, mnt^2−^), namely [(tea)_2_V_mnt_] (Figure 1; tea=tetraethylammonium) was studied as a symmetric electrolyte for nonaqueous RFBs.^36^ V_mnt_^2−^ undergoes two reversible metal‐centered one‐electron reductions and one reversible ligand‐centered one‐electron oxidation in MeCN solution.^35^ The oxidation event was charged against the first reduction event in a static H‐cell experiment, giving a 1.09 V cell with 90 % coulombic and 20 % voltaic efficiencies.^36^ In this work, we extend the application of [(tea)_2_V_mnt_] to flow cell experiments, as well as testing the wider family of bis‐mnt complexes of [(tea)Fe_mnt_], [(tea)_2_Co_mnt_], [(tea)_2_Ni_mnt_], and [(tea)_2_Cu_mnt_] as single‐species electrolytes for nonaqueous RFBs (Figure 1).

## Results and Discussion

### Synthesis

**Scheme 1:**
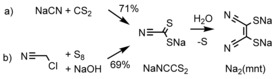
Synthetic pathways to the Na_2_mnt ligand: a) from NaCN and CS_2_ in DMF^37^, ^38^, ^39^ and b) from chloroacetonitrile, NaOH, and sulfur in DMF.^40^

The widely used synthetic method for the mnt^2−^ ligand, first reported by Bähr and Schleitzer in 1957, proceeds first by formation of the sodium cyanodithioformate (NaNCCS_2_) intermediate from sodium cyanide and carbon disulfide, followed by dimerization/desulfurization to give Na_2_mnt (Scheme 1 a).^37^, ^38^, ^39^ However, in the interests of accessing Na_2_mnt from more environmentally benign and less toxic starting materials, an alternative method reported by Hoepping and co‐workers was followed (Scheme 1 b).^40^ Here, the intermediate NaNCCS_2_ is prepared from chloroacetonitrile with NaOH and sulfur in DMF in yields identical to the more hazardous route (69 %, this work; 71 %,^39^ from NaCN+CS_2_), despite not achieving the almost quantitative yields (88–97 %) reported by Hoepping and co‐workers.^40^ The isolated intermediate was then dissolved in water and allowed to stand for 12 h to dimerize to Na_2_mnt, which was isolated by filtration to remove sulfur followed by evaporation of the filtrate to give a tan‐brown solid, in quantitative yield from NaNCCS_2_. The Na_2_mnt crude product was purified by first drying under high vacuum at 80 °C for several hours before recrystallization from EtOH/Et_2_O to give a bright‐yellow microanalytically clean powder in moderate yield (49 % from NaNCCS_2_, 34 % overall).

Anionic complexes of mnt were synthesized with tetraethylammonium (TEA^+^) cations to ensure good solubility of our electrolyte species in MeCN. Complexation reaction procedures were adapted from those previously reported to produce tris‐mnt [(tea)_2_V_mnt_],^36^, ^41^ and bis‐mnt [(tea)Fe_mnt_],^42^ [(tea)_2_Co_mnt_],^39^ [(tea)_2_Ni_mnt_],^39^ and [(tea)_2_Cu_mnt_].^39^ All were synthesized by using the corresponding metal chloride and TEACl, and recrystallized from boiling acetone/isopropanol to yield analytically clean microcrystalline solids. An assessment of the solubility of the complexes in MeCN showed that [(tea)_2_Cu_mnt_] has a relatively high solubility of 0.91 m, which is promising for high‐density RFBs with highly concentrated electrolytes, whereas [(tea)_2_V_mnt_], [(tea)_2_Co_mnt_], and [(tea)_2_Ni_mnt_] have more modest solubilities of 0.53 m, 0.39 m, and 0.30 m, respectively. [(tea)Fe_mnt_] has poor solubility in MeCN (0.03 m) and is therefore not suitable for application.

### Electrochemical properties

**Figure 2:**
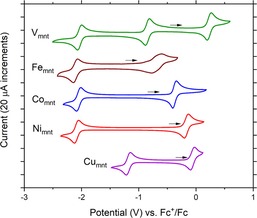
Cyclic voltammograms of 1 mm [(tea)_2_V_mnt_] (green), [(tea)Fe_mnt_] (brown), [(tea)_2_Co_mnt_] (blue), [(tea)_2_Ni_mnt_] (red), and [(tea)_2_Cu_mnt_] (purple) in 0.1 m TBAPF_6_ MeCN solution on glassy carbon electrodes at 100 mV s^−1^. Black arrows indicate the starting point and direction for voltammetry. Second voltammetry scans shown.

Cyclic voltammetry (CV) of the five complexes in relevant conditions was performed on glassy carbon to assess their suitability as candidates for nonaqueous RFB electrolytes (Figure 2, Table 1). All complexes exhibit at least one oxidation and one reduction process, making them potential electrolytes for single‐species flow batteries, that is, as both the posolyte and negolyte solutions. [(tea)_2_V_mnt_] displays two reversible reduction and one reversible oxidation events at −2.032 V, −0.849 V, and 0.230 V (vs. Fc/Fc^+^). In a RFB, a posolyte of [(tea)_2_V_mnt_] accessing the 0.230 V oxidation process could be charged against the −0.849 V reduction process in a negolyte of the same material to give a battery of *V*_cell_=1.08 V. To achieve even greater potential, charging the 0.230 V process against a (pre‐charged) negolyte reduction at −2.032 V would yield *V*_cell_=2.26 V. The bis‐mnt complexes all exhibit one reduction and one oxidation process, allowing potential use as single‐species RFB electrolytes, with cell potentials of 1.12–1.92 V (Table 1). Single‐species RFBs operating at saturated concentrations of the dithiolene complexes would therefore have quite favorable theoretical maximum energy densities of 7.7–16 Wh L^−1^, with the exception of the poorly soluble [(tea)Fe_mnt_] at 0.6 Wh L^−1^ (Table 2). Each complex also exhibits at least one irreversible oxidation process at approximately 0.5–1 V (vs. Fc/Fc^+^; see the Supporting Information, Figures S1–S5); however, in this work, the threshold potentials for charge–discharge battery cycling experiments are carefully chosen to avoid these irreversible oxidation processes.

**Table 1:** Redox potentials (vs. Fc/Fc^+^), as measured by CV at 100 mV s^−1^ on glassy carbon on 1 mm solutions of the complex in 0.1 m TBAPF_6_ MeCN solution. The theoretical cell potential, *V*_cell_=*E*_1/2_^ox^−*E*_1/2_^red^, for a symmetric flow battery of the corresponding electrolyte solution is also given.

Complex	*E*_1/2_^ox^ [V] (Δ*E* [mV])	*E*_1/2_^red1^ [V] (Δ*E* [mV])	*E*_1/2_^red2^ [V] (Δ*E* [mV])	*V*_cell_ [V]
[(tea)_2_V_mnt_]	0.230 (73)	−0.849 (69)	−2.032 (74)	1.08, 2.26^[a]^
[(tea)Fe_mnt_]	−0.689 (177)	−2.106 (77)	–	1.42
[(tea)_2_Co_mnt_]	−0.375 (61)	−2.050 (74)	–	1.68
[(tea)_2_Ni_mnt_]	−0.166 (70)	−2.088 (69)	–	1.92
[(tea)_2_Cu_mnt_]	−0.061 (69)	−1.180 (71)	–	1.12

[a] The larger cell potential for a battery employing the second reduction process of V_mnt_ as the negolyte.

**Table 2:** Solubilities in MeCN, theoretical maximum RFB energy densities, diffusion coefficients, and electrochemical rate constants.

Complex	Solubility	Energy density	Diffusion coefficient [cm^2^ s^−1^]	Rate constant [cm s^−1^]
	[mol l ^−1^]	[Wh l ^−1^]	M_mnt_^2−^→M_mnt_^1−^/M_mnt_^2−^→M_mnt_^3−^	M_mnt_^2−^→M_mnt_^1−^/M_mnt_^2−^→M_mnt_^3−^
V_mnt_^2−^	0.53	7.7, 16	8.8×10^−6^/8.6×10^−6^	1.66×10^−2^/1.53×10^−2^
Fe_mnt_^−^	0.03	0.6	–	–
Co_mnt_^2−^	0.39	8.8	1.3×10^−5^/1.1×10^−5^	1.16×10^−2^/1.35×10^−2^
Ni_mnt_^2−^	0.30	7.7	1.4×10^−5^/1.3×10^−5^	1.63×10^−2^/1.00×10^−2^
Cu_mnt_^2−^	0.91	14	9.3×10^−6^/9.0×10^−6^	1.39×10^−2^/1.45×10^−2^

All redox processes observed for the five complexes have peak separations (Δ*E*=69–77 mV, Table 1) close to that expected for a reversible one‐electron process, suggesting highly reversible redox behavior, with the exception of [(tea)Fe_mnt_]. For the Fe complex, the process at −0.689 V (vs. Fc/Fc^+^) has an abnormally large peak separation of 177 mV (Table 1) and the peaks appear much broader than for the reduction process at −2.016 V indicating sluggish electrochemical kinetics. Although the other four complexes are synthesized as dianions, the complex anion of [(tea)Fe_mnt_] is monoanionic. Attempts to improve the reversibility of the Fe complex′s redox activity by accessing the dianionic complex, that is, Fe_mnt_^2−^, were unsuccessful despite using Fe^II^ halide starting materials and working under dry, anaerobic (Schlenk) conditions. For the four complexes that display (quasi)reversible electrochemistry (M=V, Co, Ni, Cu), variable scan rate CV was conducted (Figures S6–S9). Randles–Sevcik analyses were performed to measure diffusion coefficients in the range 8.6×10^−6^–1.4×10^−5^ cm^2^ s^−1^, (Table 2), which are favorable for facile mass transport; they are comparable to those reported for high‐performing nonaqueous electrolytes based on ferrocene and cobaltacene (1.41–2.23×10^−5^ cm^2^ s^−1^),^43^ and higher than the widely studied vanadium acetylacetonate (1.8–2.9×10^−6^ cm^2^ s^−1^)^16^ and iron tris‐bipyridine (1.56×10^−6^ cm^2^ s^−1^)^25^ systems. Rotating disk electrode (RDE) studies with M=V, Co, Ni, and Cu complexes were also performed to evaluate electrochemical rate constants. Koutecký–Levich analysis of the data yielded electrochemical rate constants in the range 1.00–1.66×10^−2^ cm s^−1^ (Table 2).

### V_mnt_ battery cycling experiments

**Figure 3:**
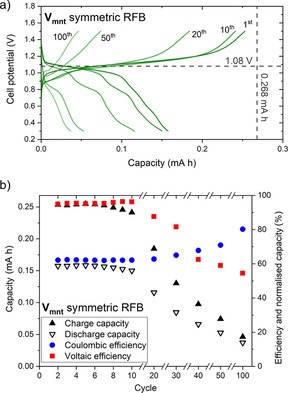
(a) Cell potential vs. capacity and (b) capacities and efficiencies for selected charge–discharge cycles at ±0.48 mA cm^−2^ constant current density for 1 mm [(tea)_2_V_mnt_] in 0.1 m TBAPF_6_ MeCN solution with Celgard separator. Dashed gray lines in (a) indicate the theoretical cell potential and capacity.

In a previous report by Cappillino and co‐workers,^36^ [(tea)_2_V_mnt_] was shown to have promising charge–discharge performance in non‐flow H‐cell experiments. Here, we anticipated improved performance in a flow cell, owing to the enhanced mass transport and decreased cell resistances arising from pumped electrolytes and smaller inter‐electrode separations, respectively. Assessment of [(tea)_2_V_mnt_] in MeCN as a symmetric electrolyte was performed in a small‐scale RFB with 2.08 cm^2^ carbon paper electrodes, a porous Celgard separator, and 10 mL of electrolyte in each half‐cell (see the Supporting Information for a full description). In total, 100 charge–discharge cycles were recorded at a constant current density of ±0.48 mA cm^−2^ with the threshold potential set to 1.5 V for charge cycles to avoid accessing the irreversible oxidation process at approximately 1 V (vs. Fc/Fc^+^), and 0.3 V for discharge (Figure 3 a). On the initial charge, a plateau from approximately 1.0–1.2 V is observed, indicating that charging is occurring about the expected cell potential for the one‐electron transfer process of 1.08 V, until a maximum capacity of 0.253 mA h, which is 94 % of the 0.268 mA h theoretical capacity for a one‐electron process. Upon discharge, a voltage plateau from approximately 1.1–1.0 V is observed, corresponding to a high voltaic efficiency (ratio of discharge to charge potential) of 95 % for cycle 2 (Figure 3 b). The discharge capacity, 0.158 mA h or 59 %, is less than the charge capacity, corresponding to a low coulombic efficiency (discharge to charge capacity ratio) of 62 % for cycle 2, resulting in an energy efficiency of 59 %. The charge–discharge behavior is consistent for ten cycles, but by the 20th cycle a second discharge plateau is observed at around 0.8 V, indicating that the battery composition has changed such that alternative redox processes are occurring. Although charge–discharge was achieved up to 100 cycles, good performance is only observed to around 20 cycles. Indeed, by operating in a flow cell, enhanced performance is achieved in comparison to the previously reported non‐flowing H‐cell charge–discharge experiments.^36^ Although the coulombic efficiency decreased from approximately 90 % to about 60–70 %, the voltaic efficiency was markedly increased from around 20–25 % to 95 %, resulting in a tripling of the energy efficiency, from about 20 % to 60 %, highlighting the importance of testing the proposed RFB electrolytes in flow conditions.

Next, we explored the effects of changing the flow cell separator as well as electrolyte solvent on the battery cycling performance. Switching the Celgard for a Fumapem F‐930 cation exchange membrane (CEM) gave a slightly diminished performance with similar discharge capacities but energy efficiencies approximately 10 % lower for F‐930 than for Celgard (Figures S12 and S13).

We attribute the poorer performance of the applied CEM to its smaller thickness (30 μm) and the undesirable physical properties of the membrane material in MeCN solvent. Presently, membranes for use in organic solvents do not yet exist and those designed for aqueous electrolyte typically display excessive swelling and fragility in some organic solvents. In contrast, Celgard, being composed of polyethylene, demonstrates superb chemical stability and mechanical properties in aggressive electrolytes. Using propylene carbonate (PC) as the electrolyte solvent appears to again give a similar performance, with slightly better coulombic and slightly worse voltaic efficiencies, resulting in energy efficiencies just below 50 % (Figures S15 and S16). We attribute the poorer battery performance to the lower conductivity and higher viscosity of PC, which results in higher cell resistances. However, again the efficiencies obscure the overall poor performance of [(tea)_2_V_mnt_] in PC as the discharge capacities are in fact much lower than in MeCN at 28 % and 59 %, respectively, on cycle 2. The large capacity fade in PC solvent occurs mostly owing to a poor first cycle performance with charge–discharge capacities being 105 %/33 % and 43 %/28 % for cycles 1 and 2, respectively.

[(tea)_2_V_mnt_] exhibits a second reversible reduction process, V_mnt_^4−/3−^, which if charged as a negolyte against a posolyte V_mnt_^2−/1−^ process, gives a RFB of *V*_cell_=2.26 V, which would have a larger theoretical energy density of 16 Wh L^−1^. To access the second reduction process in an RFB, first a battery of V_mnt_^2−^ in each half‐cell was charged to 1.5 V, to give V_mnt_^3−^ negolyte and V_mnt_^1−^ posolyte solutions. The V_mnt_^1−^ posolyte was discarded and replaced with fresh starting material, giving V_mnt_^3−^ negolyte and V_mnt_^2−^ posolyte initial solutions, which were then subjected to charge–discharge cycling up to 2.7 V. Performing this experiment in both MeCN and PC gave initial charging curves with plateaus at 2.2–2.5 V around the expected potential of 2.26 V; however, essentially zero capacity was observed upon discharge and subsequent charge cycles (Figures S14 and S17).

**Figure 4:**
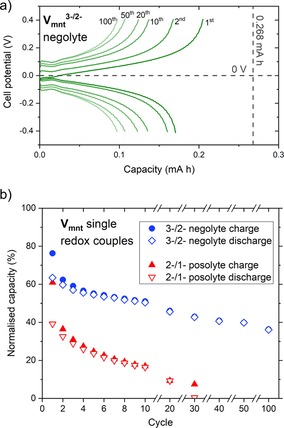
(a) Cell potential vs. capacity for the independent redox couple battery cycling of V_mnt_^3−/2−^, dashed gray lines indicate the theoretical potential and capacity. (b) Charge–discharge capacities for selected cycles of independent redox couple battery cycling of V_mnt_^3−/2−^ and V_mnt_^2−/1−^. Conditions: 1 mm [(tea)_2_V_mnt_] in 0.1 m TBAPF_6_ MeCN solution, ±0.96 mA cm^−2^ constant current density, ±0.4 V charge–discharge voltage thresholds, Celgard separator.

[(tea)_2_V_mnt_] was further investigated as a redox‐active material for nonaqueous RFBs by assessing the performance as a negolyte and as a posolyte separately by independent single redox couple cycling. Flow cell experiments were performed for both the negolyte and posolyte for 1 mm [(tea)_2_V_mnt_] in 0.1 m TBAPF_6_ (tetrabutylammonium hexafluorophosphate) MeCN solution with Celgard separator. The negolyte experiment, whereby the V_mnt_^3−/2−^ redox couple is charged/discharged, reveals good performance up to 100 cycles (Figure 4 a) with low overpotentials (≈0.1 V) and discharge capacity fading from 63 % on cycle 1 to 36 % for cycle 100 (Figure 4 b, blue data). The posolyte experiment, cycling of V_mnt_^2−/1−^ showed poorer performance compared with the negolyte, with the capacity fading much faster until almost zero capacity remained by cycle 30 (Figure 4 b, red data). These independent battery cycling data for the negolyte and posolyte solutions of [(tea)_2_V_mnt_] show that in the symmetric RFB the posolyte, that is, the V_mnt_^2−/1−^ redox process, is less stable and is mostly responsible for the significant capacity fade observed by cycle 50 (Figure 3).

**Figure 5:**
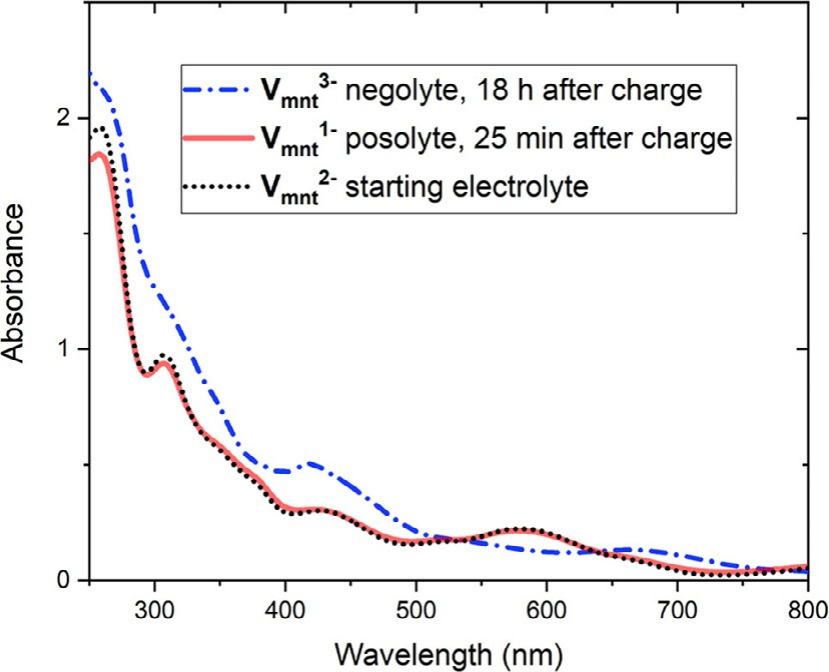
UV/Vis spectra of the V_mnt_^3−^ negolyte solution recorded 18 h after an initial battery charge cycle, the V_mnt_^−^ posolyte solution recorded 25 min after an initial battery charge cycle, and the uncharged starting V_mnt_^2−^ electrolyte solution. All solutions are 1 mm electrolytes in 0.1 m TBAPF_6_ MeCN solution diluted with MeCN by a factor of 20 to 50 μm V_mnt_^*n*−^ in 5 mm TBAPF_6_.

For each of the M=V, Co, Ni, Cu complexes, the posolyte and negolyte solutions were extracted from the symmetric RFB charge–discharge experiments after the first charge cycle and measured by UV/Vis spectroscopy to observe their stability over time. For [(tea)_2_V_mnt_], the charged negolyte solution gives a distinct spectrum from the uncharged electrolyte, most notably with the absence of the peaks at 308 and 580 nm, and appears to be stable with minimal change in the spectrum over 18 h (Figure 5). The posolyte V_mnt_^1−^ also gives a distinct spectrum immediately after the initial charge cycle, with the loss of the 308 nm peak and an increase in intensity and shift to lower wavelength of the 580 nm peak (Figure S35). However, over 25 min the spectrum evolves, most notably with the reappearance of the peak at 308 nm, to give an almost identical spectrum to that of the initial uncharged V_mnt_^2−^ electrolyte (Figure 5). These data are in agreement with the symmetric single‐redox couple flow cell data (Figure 4), with the V_mnt_^3−^ negolyte being more stable than the V_mnt_^1−^ posolyte. Furthermore, the spectra reveal that the posolyte solution is self‐discharging to the initial V_mnt_^2−^ dianion, and that this process occurs over a short time frame outside of the battery cycling environment. The mechanism for the self‐discharge of the V_mnt_^1−^ species is unclear; however, it is evident that a reducing agent (possibly trace water) must be present in the electrolyte to chemically reduce V_mnt_^1−^.

### Co_mnt_ battery cycling experiments

**Figure 6:**
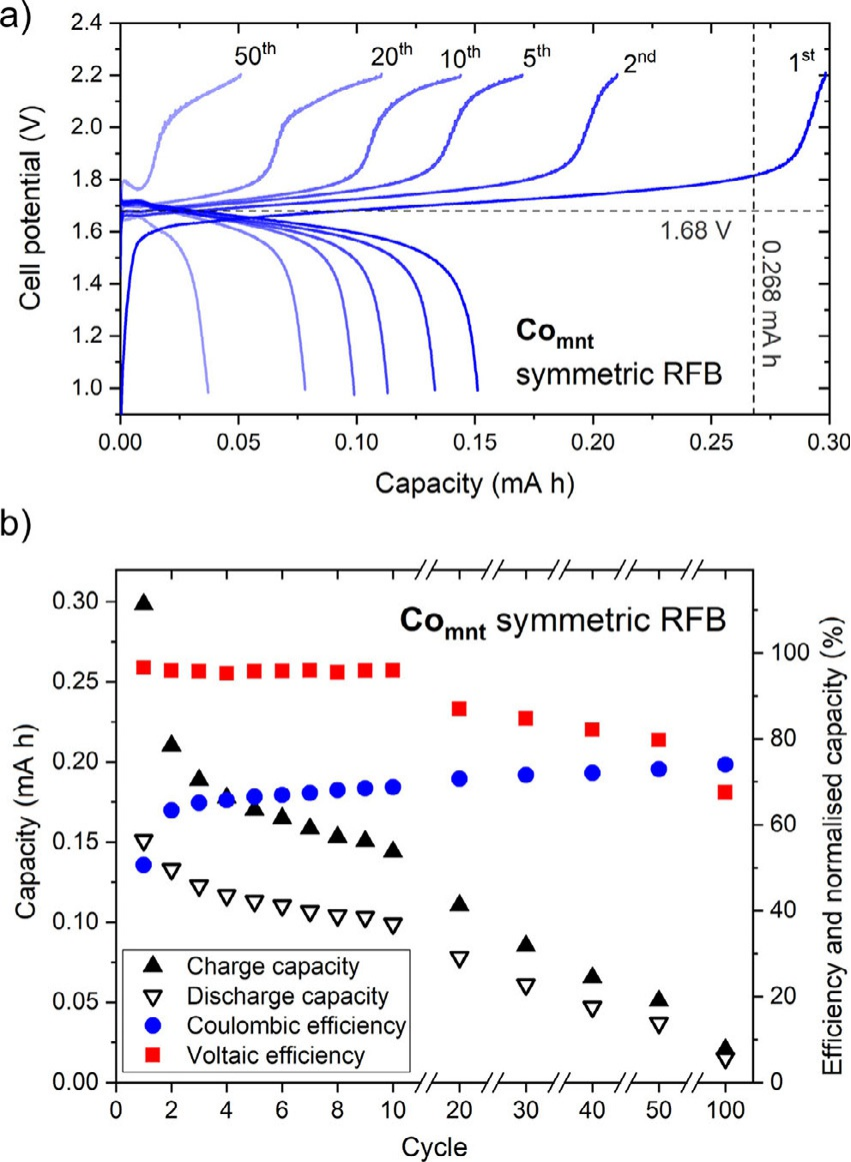
(a) Cell potential vs. capacity for selected charge–discharge cycles for [(tea)_2_Co_mnt_]. Dashed lines indicate the theoretical cell potential and capacity. (b) Capacities and efficiencies for selected charge–discharge cycles for [(tea)_2_Co_mnt_]. Conditions: 1 mm [(tea)_2_Co_mnt_] in 0.1 m TBAPF_6_ MeCN solution, Celgard separator, ±0.48 mA cm^−2^ constant current density, 2.2/1.0 V potential thresholds.

[(tea)_2_Co_mnt_] charge–discharge cycling revealed voltage plateaus centered around the expected *V*_cell_ of 1.68 V and excellent voltaic efficiencies of 96 % for the first ten cycles (Figure 6). Coulombic and energy efficiencies were 63–69 % and 61–66 % over the first ten cycles, with discharge capacity fading from 56 % to 37 %. Despite good performance initially, the discharge capacity faded steadily to 14 % on cycle 50, and to essentially zero capacity (6 %) by the 100th cycle (Figure 6). In addition, an unexpected second plateau near the 2.2 V threshold was observed on charge, which became more prominent with cycling, indicating that the battery chemistry evolved with increasing cycle number.

In the independent Co_mnt_^3−/2−^ negolyte single redox couple battery cycling, there is steady, almost complete, capacity fade over 50 cycles from 38 % to 1 % (Figures S27 and S29). The posolyte in this case, that is, the Co_mnt_^2−/1−^ single redox couple, appears more stable to multiple charge–discharge cycles, with almost zero overpotential, and a capacity fade from 75 % to 54 % over 50 cycles (Figures S28 and S29).

UV/Vis spectra of [(tea)_2_Co_mnt_] (Figures S36–S38) indicate that the charged Co_mnt_^3−^ negolyte solution is unstable, giving an almost identical spectrum to the initial Co_mnt_^2−^ solution after only a few minutes. The posolyte Co_mnt_^1−^ species is much more robust in solution, giving a distinct UV/Vis spectrum, which is almost unchanged over 22 h. This is in line with the symmetric single redox couple battery cycling data, for which the posolyte‐only Co_mnt_^2−/1−^ battery significantly outperformed the negolyte‐only Co_mnt_^3−/2−^ system.

### Ni_mnt_ battery cycling experiments

Battery cycling of [(tea)_2_Ni_mnt_] reached only small capacities of 23–32 % on charge for the first five cycles and only small voltage plateaus around the expected *V*_cell_ of 1.92 V were observed (Figure S19). Upon discharge, the cell potential steadily decreased, with no plateau, to the lower threshold potential of 1 V with <15 % capacity. Repeating the experiment in PC solvent resulted in a great improvement in battery cycling performance (Figures S20 and S21), with the initial charge cycle showing a long plateau around 1.92 V up to a capacity of 97 %. The first cycle resulted in a large capacity fade, with a discharge capacity of 41 %.

**Figure 7:**
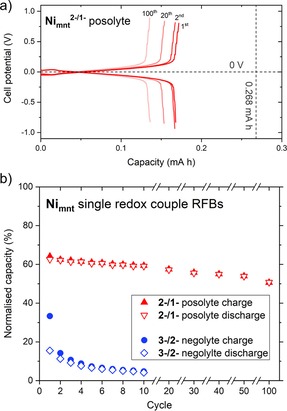
(a) Cell potential vs. capacity for the independent redox couple battery cycling of Ni_mnt_^2−/1−^, dashed lines indicate the theoretical potential and capacity. (b) Charge–discharge capacities for selected cycles of independent redox couple battery cycling of Ni_mnt_^3−/2−^ and Ni_mnt_^2−/1−^. Conditions: 1 mm [(tea)_2_Ni_mnt_] in 0.1 m TBAPF_6_ MeCN solution, ±0.48 mA cm^−2^ constant current density, ±0.8 V potential thresholds, Celgard separator.

The independent redox couple RFB experiments of [(tea)_2_Ni_mnt_] in MeCN are perhaps the most insightful, with the negolyte retaining almost no capacity and the posolyte showing a very robust performance with respect to long‐term cycling (Figure 7). The Ni_mnt_^3−/2−^ negolyte system shows a poor initial discharge capacity of 16 %, which rapidly fades to 4 % after just ten cycles (Figure 7 b, blue data), indicating that [(tea)_2_Ni_mnt_] is unstable as the negative electrolyte, and would account for the very poor battery cycling performance of the MeCN symmetric RFB, which displayed almost zero discharge capacity on the first cycle (Figure S19). In contrast, the Ni_mnt_^2−/1−^ posolyte system is very stable to charge–discharge cycling with a capacity fade from 66 % to 51 % over 100 cycles (Figure 7). Despite [(tea)_2_Ni_mnt_] being shown to be ineffective as a single‐species electrolyte in MeCN for symmetric RFBs, the Ni_mnt_^2−/1−^ redox couple appears to be very stable over multiple charge–discharge cycles, so could be utilized as a posolyte material in an asymmetric RFB.

UV/Vis spectra of charged electrolyte solutions of [(tea)_2_Ni_mnt_] (Figures S39–S41) indicate that the charged Ni_mnt_^3−^ negolyte solution is very unstable, giving an almost identical spectrum to the starting electrolyte Ni_mnt_^2−^ solution after only a few minutes. The reduced species Ni_mnt_^3−^ in MeCN and 1,2‐dimethoxyethane solutions have previously been observed to be air‐sensitive and unstable in solution^44^—indicating that in our battery system, even trace amounts of oxygen may be causing rapid discharge of the Ni_mnt_^3−^ species to the starting Ni_mnt_^2−^ state resulting in very small capacity retention for the symmetric cell. Despite this, the posolyte Ni_mnt_^1−^ species is far more stable in solution, giving a distinct UV/Vis spectrum, which shows little change over 22 h.

### Cu_mnt_ battery cycling experiments

[(tea)_2_Cu_mnt_] was tested under 100 charge–discharge cycles at ±0.48 mA cm^−2^ constant current density in MeCN with a either a Celgard separator or Fumapem F‐930 cation exchange membrane (Figures S22–S25). The initial charge cycle with Celgard reached a capacity of 0.358 mA h (134 %), suggesting that self‐discharge occurred during charging. The charge–discharge cycles exhibit voltage profiles with plateaus slightly above and below *V*_cell_=1.12 V at 1.1–1.3 V and 1.1–0.85 V on charge and discharge, respectively, giving a consistent voltaic efficiency of 85 % over 100 cycles (Figures S22 and S23). The coulombic efficiency and hence also energy efficiency remain consistent at 60–68 % and 51–58 %, respectively, over cycles 2–100. The overall energy efficiency of this system is comparable to [(tea)_2_V_mnt_] and [(tea)_2_Co_mnt_] under the same conditions, however, the capacity retention of [(tea)_2_Cu_mnt_] is far superior, with the discharge capacity only fading from 65 % to 53 % from cycle 1 to 50, and retaining 43 % at the 100th cycle. Moreover, the excellent solubility of [(tea)_2_Cu_mnt_] in MeCN, 0.91 m, make this complex the highest performing complex studied here. Using the Fumapem F‐930 cation exchange membrane, under otherwise identical conditions, resulted in much poorer battery cycling performance for the [(tea)_2_Cu_mnt_] system (Figures S24 and S25), with energy efficiencies of 38–33 % for cycles 2–10 and rapid capacity fade with a discharge capacity of only 0.7 % on cycle 20.

For [(tea)_2_Cu_mnt_], as is seen with the other bis‐mnt complexes [(tea)_2_Co_mnt_] and [(tea)_2_Ni_mnt_], the negolyte is less stable with a poor discharge capacity of 23 % and steady fade over 100 cycles to 9 % (Figures S31 and S33). Despite this, the [(tea)_2_Cu_mnt_] posolyte single redox couple battery, that is, cycling of Cu_mnt_^2−/1−^ shows a more stable performance (Figures S32 and S33), with discharge capacities approximately twice that of the negolyte over the first ten cycles before fading to a similar capacity by cycle 100.

For [(tea)_2_Cu_mnt_], both the posolyte and negolyte solutions give distinct spectra to the initial electrolyte and are stable for at least a few minutes (Figures S42–S44). However, both charged electrolytes appear to self‐discharge over the course of 24 h to give spectra resembling that of the initial Cu_mnt_^2−^ electrolyte—this is unsurprising given that both the posolyte and negolyte have similar discharge capacities after 100 cycles in the independent single redox couple battery cycling experiments. Unlike the M=V, Co, Ni complexes, which each had one of either the posolyte or negolyte observed by UV/Vis to rapidly self‐discharge, both posolyte and negolyte solutions of [(tea)_2_Cu_mnt_] are initially stable, allowing 100 charge–discharge cycles in the symmetric RFB with low capacity fade.

### Comparison of M_mnt_ battery cycling performances

**Figure 8:**
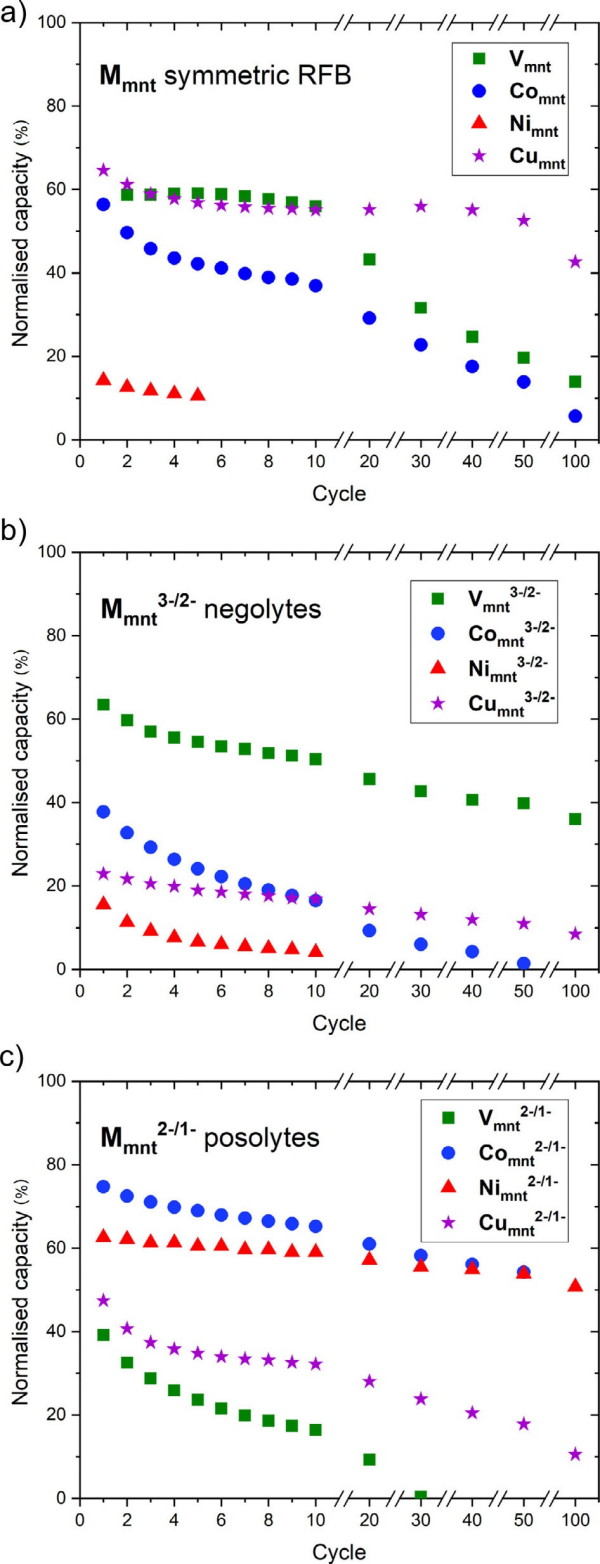
Normalized discharge capacities for selected cycles of RFB experiments of [(tea)_2_M_mnt_] complexes in MeCN with Celgard separator: (a) Symmetric full‐cell cycling, (b) independent single redox couple M_mnt_^3−/2−^ negolytes, (c) independent single redox couple M_mnt_^2−/1−^ posolytes.

In a symmetric full‐cell RFB, [(tea)_2_V_mnt_] and [(tea)_2_Co_mnt_] showed modest performances, with the initial ten cycles displaying high voltaic efficiencies of 95–96 % each; however, long‐term cycling was not possible with significant discharge capacity fade to 20 % and 14 %, respectively, by cycle 50 (Figure 8 a). [(tea)_2_Cu_mnt_] displays a comparable performance over ten charge–discharge cycles, however, it shows superior capacity retention with a smaller capacity fade to 43 % over 100 cycles (Figure 8 a), and is the best performing symmetric RFB studied here. For [(tea)_2_Ni_mnt_], which displayed the largest cell potential for a simple one‐electron disproportionation symmetric electrolyte, almost zero discharge capacity could be achieved in MeCN (Figure 8 a); however, a switch to propylene carbonate solvent did allow for charge–discharge cycling, albeit with poor performance. Battery cycling of [(tea)Fe_mnt_] returned almost zero capacity upon discharge in the first cycle, and then failed to hold any significant capacity upon recharging (Figure S18), possibly arising from the poor reversible behavior of the redox process at −0.689 V (vs. Fc/Fc^+^). In addition, the plateau voltage at approximately 0.9–1.2 V is well below the expected *V*_cell_ of 1.42 V, indicating that alternative redox processes are occurring during the charge cycle.

Independent single redox couple flow cell experiments revealed that for [(tea)_2_V_mnt_] the negolyte V_mnt_^3−/2−^ is more robust than the posolyte V_mnt_^2−/1−^ with greater capacity retention achieved for the negolyte‐only experiment (Figure 8 b and c), despite both processes being reversible on a CV timescale. A previous study on the V_mnt_ redox series, using X‐ray absorption spectroscopy and DFT calculations, determined that oxidation of V_mnt_^2−^=[V^IV^(mnt_3_^6−^)]^2−^ is a ligand‐centered process to the diradical [V^IV^(mnt_3_^5−.^)]^−^=V_mnt_^1−^ species and involves a change in geometry from distorted octahedral to trigonal prismatic, whereas reduction to V_mnt_^3−^ is metal‐centered, that is, V^IV^ to V^III^.^35^ Although no conclusions on the stability of the redox products were made in the above report, and both redox processes are reversible by CV,^35^ two different processes (ligand‐ vs. metal‐centered redox) appear to give RFB electrolytes of contrasting stability.

Conversely, for the square‐planar bis‐mnt complexes (M=Co, Ni, Cu), it was the posolyte that showed greater capacity retention over multiple charge–discharge cycles of the single redox couple, whereas the negolyte in each case was unstable (Figure 8 b and c). A previous study, in THF solution, found that metal‐centered reduction of Co_mnt_^2−^ gives the air‐sensitive unstable Co_mnt_^3−^ trianion, whereas ligand‐centered oxidation to the Co_mnt_^1−^ monoanion is more robust.^45^ Similarly, for Ni_mnt_^2−^, reduction is metal‐centered and the Ni_mnt_^3−^ product has been observed to be unstable in air, reverting back to the dianion.^44^ Trace amounts of oxygen present in the solvent, despite working under glovebox conditions, could well be responsible for the rapid capacity fade of the negolytes of the Co_mnt_ and Ni_mnt_ RFBs. Another previous dithiolene study observed that weak protic acids oxidize the basic and strongly reducing Co_mnt_^3−^ and Ni_mnt_^3−^ species in THF solution, either directly to M_mnt_^2−^, or through protonation to [M(H)(mnt)_2_]^2−^ hydrides followed by subsequent decomposition to M_mnt_^2−^ and H_2_;^46^ this could be another possible mechanism for the self‐discharge of Co_mnt_^3−^ and Ni_mnt_^3−^ negolytes observed by UV/Vis in the present study. More recently, sulfur K‐edge X‐ray absorption spectroscopy has been used to determine Ni_mnt_^2−^ oxidation to Ni_mnt_^1−^ is ligand‐centered,^34^ and results in a very stable Ni_mnt_^2−/1−^ posolyte system in the present study. Again, we note a contrast between ligand‐ versus metal‐centered redox processes and the stability of the resulting RFB electrolytes; however, conversely to six‐coordinate tris‐mnt V_mnt_ where metal‐centered reduction gives a stable negolyte, for four‐coordinate bis‐mnt Co_mnt_ and Ni_mnt_ it is ligand‐centered oxidation that results in the more stable posolytes. The posolyte of Cu_mnt_ displays poorer capacity retention than the posolytes of Co_mnt_ and Ni_mnt_ (Figure 8 c); interestingly, oxidation of Cu_mnt_^2−^ to Cu_mnt_^1−^ is a metal‐centered Cu^II^ to Cu^III^ process,^34^ in contrast to ligand‐centered oxidation of Co_mnt_^2−^ and Ni_mnt_^2−^.

The best performing electrolytes are the [(tea)_2_V_mnt_]^3−/2−^ negolyte (Figure 8 b), and the [(tea)_2_Co_mnt_]^2−/1−^ and [(tea)_2_‐ Ni_mnt_]^2−/1−^ posolytes (Figure 8 c), which retain approximately 40–50 % capacity after 100 charge–discharge cycles and are therefore promising for use in asymmetric RFBs.

## Conclusions

Five transition‐metal complexes of the dithiolene ligand mnt have been assessed for application as redox‐active materials for single‐species electrolytes in symmetric nonaqueous RFBs. The V, Co, Ni, and Cu complexes all exhibit at least two reversible redox‐couples and have favorable diffusion coefficients/electrochemical rate constants suitable for RFB charge–discharge battery cycling in MeCN solvent. Although [(tea)_2_Ni_mnt_] and [(tea)Fe_mnt_] MeCN electrolytes displayed poor performance in symmetric RFBs, [(tea)_2_V_mnt_], [(tea)_2_Co_mnt_], and [(tea)_2_Cu_mnt_] were able to be charged/discharged for up to 100 cycles with high voltaic efficiencies. However, capacity retention over multiple cycles proved challenging, with 43 % retention being the best achieved over 100 cycles for [(tea)_2_Cu_mnt_].

Analyzing the negolyte and posolyte solutions separately, by independent single redox couple “0 V” battery cycling, revealed that in each case one oxidation state was far more robust for long‐term cycling. This was especially insightful for [(tea)_2_Ni_mnt_]_,_ which could not be charged/discharged in a symmetric MeCN RFB; the negolyte suffers immediate capacity fade, whereas the posolyte is robust for 100 charge–discharge cycles. Monitoring of the UV/Vis spectra of freshly charged negolyte and posolyte solutions of each complex over time agreed with the observations of posolyte/negolyte stability from the single redox couple RFB experiments, and indicated that unstable redox states were self‐discharging to the starting dianionic state. The self‐discharging mechanism results in a cell imbalance, inherently limiting the capacity of the symmetric RFBs. Future work will target derivatized dithiolene ligands to increase the stability of the oxidized and reduced states of the complexes.

Although the dithiolene complexes studied here are not suitable as materials for single‐species symmetric RFBs, longer‐term stability was observed for [(tea)_2_V_mnt_]^3−/2−^ negolyte and [(tea)_2_Co_mnt_]^2−/1−^ and [(tea)_2_Ni_mnt_]^2−/1−^ posolyte solutions and are therefore applicable as single electrolytes in asymmetric RFBs. The development of ion‐selective membranes that are suitable for use with nonaqueous electrolytes is at present a limitation in the field of nonaqueous RFBs and as such hinders the use of the studied dithiolene metal complexes in asymmetric battery designs.

## Experimental Section

### Synthesis

All compounds were synthesized according to previously reported methods,^36^, ^39^, ^40^, ^41^, ^42^ albeit with modifications (see the Supporting Information for full details).

### Voltammetry

Voltammetry experiments were performed in anaerobic 1 mm solutions of each complex, with 0.1 m TBAPF_6_ supporting electrolyte, in HPLC grade acetonitrile. All voltammetry experiments were performed under an inert N_2_ atmosphere; solutions were fully purged before use and a N_2_ headspace was maintained throughout the experiments. Cyclic voltammetry (CV) was performed by using a standard 20 mL 3‐electrode glass cell (BASi®) consisting of a platinum wire auxiliary electrode, Ag/AgPF_6_ quasi‐reference (Ag wire in a glass fritted tube of 0.1 m TBAPF_6_ in MeCN), and a glassy carbon (GC) disk working electrode (3.0 mm diameter, BASi, Alvatek, UK). Redox‐couple reversibility and diffusion coefficients, calculated by Randles–Sevcik analysis, were assessed by variation of the scan rate. Rotating disk electrode (RDE) studies were performed by using a 60 mL RRDE‐3A apparatus (ALS Co., Ltd) with a 5 mm diameter GC working electrode at rotation rates in the range 300–3000 rpm. Electrochemical rate constants were derived from the RDE data by Koutecký–Levich analysis. Working electrodes were polished before use with two grades of diamond slurries (3 μm and 0.25 μm, Buehler) and alumina suspension (0.05 μm, Buehler) prior to sonication in deionized water, acetone rinsing, and air drying. Redox potentials were reported against the ferrocene/ferrocenium ion redox couple as an internal standard, except for variable scan rate studies, which are reported against the Ag^+^/Ag quasi reference (see the Supporting Information). Measurements were recorded by using a PC‐controlled Emstat (PalmSens) with a resolution of 1 mV.

### Flow battery charge–discharge experiments

Galvanostatic battery experiments were performed by using a conventional zero‐gap flow‐cell manufactured in house; the “Gen 2 flow‐cell” was reproduced from a reported method^47^, ^48^ (see the Supporting Information for further details). Experiments were conducted by using a flow‐through flow field (FTFF), 1 mm carbon paper electrodes (Technical Fibre Products Ltd., polyvinyl alcohol binder, 2.08 cm^2^ active area) and either a Celgard membrane (Celgard® 2500 Microporous Membrane, 25 μm thickness) or F‐930 cation exchange membrane (fumapem® F‐930, FuMA‐Tech GmbH, 30 μm thickness). Battery experiments were conducted with 10 mL half‐cell solutions (20 mL total volume) of 1 mm redox material in 0.1 m TBAPF_6_ (TCI chemicals) in either MeCN (99.9 %, extra dry, over molecular sieves, AcroSeal™, ACROS Organics™) or propylene carbonate (99.5 %, anhydrous, AcroSeal™, ACROS Organics™) at a flow‐rate of 10 mL min^−1^ by use of a Masterflex L/S peristaltic pump (Cole‐Parmer). Experiments were conducted within a N_2_ glovebox (Saffron Scientific Ltd. or MBRAUN), which was maintained with oxygen and water levels at a maximum of 1 ppm. Charge cycles were performed at constant current density until the defined upper and lower potential thresholds were reached. The same magnitude of current was used upon both the charge and discharge. Charge–discharge cycling was controlled by either an Autolab (Metrohm AG) or Compactstat (Ivium Technologies) potentiostat. For the single redox couple “0 V” experiments, the following example procedure was performed; to examine the negolyte, a symmetric flow cell with M_mnt_^2−^ initial starting electrolyte in each half‐cell was charged at constant current to access the M_mnt_^3−^ as the negolyte and M_mnt_^−^ as the posolyte. The posolyte was then replaced with fresh M_mnt_^2−^ electrolyte, before battery cycling at constant current between upper/lower potential thresholds just above/below 0 V. To examine the posolyte, the M_mnt_^3−^ negolyte is instead replaced with fresh M_mnt_^2−^ electrolyte after the initial electrolysis.

### UV/Vis spectroscopy

UV/Vis spectra were recorded with an Agilent Cary 60 spectrophotometer. Spectra of the as‐synthesized materials were recorded for 50 μm solutions in MeCN. Spectra of charged electrolytes were recorded from solutions prepared as follows: in a N_2_‐filled glovebox, an initial charge cycle was first performed on a solution of 1 mm complex in 0.1 m TBAPF_6_ MeCN solution in each half‐cell. Next, 50 μm solutions of each charged electrolyte were prepared by extracting 250 μL from the battery electrolytes and diluting to 5 mL (5 mm TBAPF_6_ supporting salt concentration). The solutions were transferred to sealed quartz cuvettes (Starna Scientific, 1 cm path length), removed from the glovebox, and the UV/Vis spectra were recorded immediately. The time of *t*=0 presented in the results represents approximately 5 min after the initial charge cycle of the flow cell was completed.

### Solubility measurements

The solubility of each complex in pure MeCN was measured by UV/Vis spectroscopy. The absorbance of stock solutions of the complex at 10, 20, 30, 40, and 50 μm was measured at the following wavelengths: [(tea)_2_V_mnt_], 258.5 nm; [(tea)Fe_mnt_], 241.0 nm; [(tea)_2_Co_mnt_], 262.5 nm; [(tea)_2_Ni_mnt_], 270.5 nm; [(tea)_2_Cu_mnt_], 281.0 nm. In each case, a Beer–Lambert calibration relating concentration and absorbance (*R* ^2^>0.999) was achieved and used to calculate the concentration of the unknown solutions. A saturated solution of each complex was prepared by making a suspension of complex (150–300 mg) in MeCN (0.3–0.5 mL), which was sonicated for at least 1 h, then quickly filtered through cotton wool, and series‐diluted into the calibration range by taking 20 μL into 20 mL MeCN (200‐fold dilution), then 250 μL into 10 mL MeCN (40‐fold dilution).

## Conflict of interest

*The authors declare no conflict of interest*.

## Supporting information

As a service to our authors and readers, this journal provides supporting information supplied by the authors. Such materials are peer reviewed and may be re‐organized for online delivery, but are not copy‐edited or typeset. Technical support issues arising from supporting information (other than missing files) should be addressed to the authors.

Supplementary
