# Supplementary material for: Dithiolene Complexes of First‐Row Transition Metals for Symmetric Nonaqueous Redox Flow Batteries
Source: ChemSusChem. 2019 Sep 3;12(19):4506–15. doi: 10.1002/cssc.201901702 (PMC6852480; doi:10.1002/cssc.201901702)
Supplement: Supplementary file 1 — Supplementary [file CSSC-12-4506-s001.pdf]

CHEMISTRY & SUSTAINABILITY
CHEMSUSCHEM
ENERGY & MATERIALS

Supporting Information

Dithiolene Complexes of First-Row Transition Metals for Symmetric Nonaqueous Redox Flow Batteries

Ross W. Hogue, Craig G. Armstrong, and Kathryn E. Toghill*[a]

cssc_201901702_sm_miscellaneous_information.pdf

Synthesis

General synthesis details

All chemicals were used as received from the supplier without further purification. NMR spectra were recorded on a Bruker Ultrashield 400 Plus spectrometer at 298 K. CHNS elemental analyses were performed on an Elementar vario MICRO cube. High resolution mass assignment was performed using a Shimadzu LCMS-IT-TOF with electrospray ionisation (ESI).

Disodium *cis*-1,2-dicyanoethylene-1,2-dithiolate (Na_2mnt). The synthesis is adapted from that previously described,[1] with modifications. To a suspension of NaOH (10.17 g, 254 mmol) in 30 mL DMF was slowly added sulphur (8.15 g, 254 mmol) over 40 min. The dark red reaction mixture was cooled to 0°C and a solution of chloroacetonitrile (6.392 g, 84.7 mmol) in 6 mL DMF was added dropwise over 1 h. The tan-brown reaction mixture was allowed to slowly warm to room temperature and was stirred for 1.5 h. Isopropanol (50 mL) was added and the reaction warmed to ~60°C, hot filtered, and washed with 40 mL of boiling isopropanol. To the cooled filtrate was added 100 mL of diethyl ether, and the solution was cooled to -20°C. Scratching the flask with a spatula caused crystallisation of the sodium cyanodiformate intermediate (·3DMF solvate, $NCCS_2Na$·3DMF, 20.1 g, 69%), which was isolated by filtration, washed with 100 mL diethyl ether, and air dried. $NCCS_2Na$·3DMF was dissolved in 170 mL H_2O to give a brown solution which was left to stand overnight, in which time dimerization of the intermediate $NCCS_2Na$·3DMF results in a light brown solution with a sulphur precipitate. The mixture was filtered through celite and washed with 50 mL H_2O. Sulfur immediately started to precipitate in the filtrate so this was left to stand for a further three days to allow the dimerization to go to completion. The solution was again filtered through celite, washed with 50 mL H_2O. The light brown filtrate was dried to a brown oil by rotary evaporator, taken up in 100 mL EtOH and again evaporated to give a tan-brown powder, which was further dried under hi-vacuum at 80°C for 5 h to yield 6.8 g (approx. quantitative from $NCCS_2Na$·3DMF) of crude product. This was suspended in 40 mL boiling EtOH, hot filtered, and washed with 40 mL boiling EtOH. To the cooled filtrate was added 50 mL diethyl ether, and cooled to 0°C, to produce a bright yellow powder precipitate, which was filtered and washed with 100 mL of diethyl ether, then dried under hi-vacuum to yield **Na_2mnt·1¾H_2O** (2.978 g, 14.27 mmol, 34%). ^{13}C NMR (D_2O, 100 MHz): δ = 126.30, 122.74. Anal calcd. for $C_4N_2Na_2S_2$·1¾H_2O: C 22.07, H 1.62, N 12.87, S 29.46%; found: C 22.34, H 1.48, N 12.63, S 29.47%. Repeated syntheses returned yields of 11-34% with solvent water content of ·¾H_2O to ·3H_2O. ^{13}C NMR data consistent with that reported in the literature.[2]

$(TEA)_2[V(mnt)_3]$·½H_2O = $(TEA)_2V_{mnt}$. The synthesis of $(TEA)_2[V(mnt)_3]$ has been described previously,[3] however was synthesised here by an adapted procedure for the related $[(C_6H_5)_4As]_2[V(mnt)_3]$ complex.[4] To a yellow suspension of Na_2mnt·3H_2O (1.11 g, 4.62 mmol) in 20 mL dry THF was added solid VCl_3 (242 mg, 1.54 mmol) resulting in a dark green suspension. Upon stirring for 40 min the reaction mixture was a green solution, and a solution of tetraethylammonium chloride in 2 mL of EtOH was added dropwise. After stirring overnight, the solution was filtered and washed with 20 mL dry THF and the filtrate evaporated to dryness, washed with 2-propanol and the residual green solid taken up in 5 mL hot acetone, and 15 mL boiling isopropanol then cooled on ice. Black microcrystalline material was isolated by filtration, then recrystallized by dissolving in 5 mL acetone and 10 mL isopropanol, then reduced in volume to 10 mL by heating. Black needle crystals were filtered and dried in air then under high vacuum yielding **$(TEA)_2[V(mnt)_3]$·½H_2O** 315 mg, 0.43 mmol, 28%. Anal calcd. for $C_{28}H_{40}N_8S_6V$·½H_2O: C 45.38, H 5.58, N 15.12, S 25.96%; found: C 45.54, H 5.51, N 15.11, S 26.32%; HRMS (ESI$^-$): m/z = 600.9548 {$(TEA)[V(mnt)_3]^-$ (calcd = 600.9550), 235.3978 $[V(mnt)_3]^{2-}$ (calcd =

235.3980). UV-vis: λ_{max} / cm (ε / L mol^{-1} cm^{-1}) = 223.5 (40187), 258.5 (39152), 307.5 (19508), 427.5 (6038), 579.5 (4475).

(TEA)[Fe(mnt)$_2$] = (TEA)Fe$_{mnt}$. The synthesis is adapted from that previously described with modifications.[5] To a yellow solution of Na_2mnt·1¾H_2O (648 mg, 2.98 mmol) in 20 mL 1:1 H_2O/EtOH was added a solution of $FeCl_3$ (242 mg, 1.49 mmol) in 1.5 mL H_2O resulting immediately in a dark brown-red solution. The reaction was stirred for 30 min, filtered through celite and washed with 10 mL 1:1 H_2O/EtOH. To the filtrate was added a 4 mL EtOH solution of TEACl (494 mg, 2.98 mmol) dropwise to give a dark precipitate. After stirring for 30 min, the sticky black solid mass was filtered, washed with 10 mL water then 10 mL ice cold 1:1 H_2O/EtOH, then recrystallized by dissolving in 15 mL hot acetone, adding 20 mL hot isopropanol then reducing the volume to 20 mL by heating, and cooling to 0°C. The resulting black microcrystalline solid was filtered, washed with 20 mL ice cold isopropanol, and dried in air then in vacuo to yield **(TEA)[Fe(mnt)$_2$]** (358 mg, 0.77 mmol, 52%). Anal calcd. for $C_{16}H_{20}FeN_5S_4$: C 41.20, H 4.32, N 15.01, S 27.50%; found: C 40.83, H 4.23, N 15.09, S 27.54%; HRMS (ESI$^-$): m/z = 335.8354 [Fe(mnt)$_2$]$^-$ (calcd = 335.8361). UV-vis: λ_{max} / cm (ε / L mol^{-1} cm^{-1}) = 241.0 (19904), 270.0 (26699), 300.0 (15561), 358.5 (12060), 451.0 (9118).

(TEA)$_2$[Co(mnt)$_2$] = (TEA)$_2$Co$_{mnt}$. The synthesis is adapted from that previously described with modifications.[6] Under an atmosphere of N_2 Na_2mnt·1¼H_2O (400 mg, 1.92 mmol) was dissolved 1:1 H_2O/EtOH (6mL, degassed with N_2) to give a bright yellow solution. A purple solution of $CoCl_2·6H_2O$ (228 mg, 0.96 mmol) in 1:1 H_2O/EtOH (3mL, degassed with N_2) under N_2 was added to the Na_2mnt solution via syringe, causing a colour change to dark red. After stirring for 30 min, a solution of TEACl (319 mg, 1.92 mmol) in 1:1 H_2O/EtOH (2mL, degassed with N_2) under N_2 was added via syringe giving an immediate black precipitate. After stirring for 20 min, the precipitate was filtered under a stream of N_2, washed with ice cold 1:1 H_2O/EtOH and dried under hi-vacuum to yield **(TEA)$_2$[Co(mnt)$_2$]·½H_2O** (397 mg, 0.65 mmol, 68%) as a black microcrystalline solid. Anal calcd. for $C_{24}H_{40}CoN_6S_4$·½H_2O: C 47.35, H 6.79, N 13.80, S 21.06%; found: C 47.35, H 6.70, N 13.86, S 21.42%; HRMS (ESI$^-$): m/z = 338.8339 [Co(mnt)$_2$]$^-$ (calcd = 338.8343). UV-vis: λ_{max} / cm (ε / L mol^{-1} cm^{-1}) = 233.5 (25897), 262.5 (32615), 316.0 (20708), 416.0 (7328), 458.5 (6015), 549 (3128).

(TEA)$_2$[Ni(mnt)$_2$] = (TEA)$_2$Ni$_{mnt}$. The synthesis is adapted from that previously described with modifications.[6] To a yellow solution of Na_2mnt·1¼H_2O (812 mg, 3.89 mmol) in 15 mL 1:1 H_2O/EtOH was added a green solution of $NiCl_2·6H_2O$ (462 mg, 1.94 mmol) in 2 mL 1:1 H_2O/EtOH resulting immediately in a red solution. After stirring for 20 min a 2 mL solution of TEACl (806 mg, 4.86 mmol) in 1:1 H_2O/EtOH was added dropwise to give an orange precipitate. The reaction was stirred for a further 40 min, filtered and washed with 10 mL H_2O then 4 mL ice cold 1:1 H_2O/EtOH and air dried. The collected orange solid is then dissolved in 12 mL hot acetone and 20 mL of isopropanol is added slowly with heating to boiling, then the red solution is cooled to 0°C. Dark red crystals were filtered, washed with 10 mL ice-cold isopropanol, then dried in vacuo at 60°C to yield **(TEA)$_2$[Ni(mnt)$_2$]** (918 mg, 1.53 mmol, 79%). Anal calcd. for $C_{24}H_{40}N_6NiS_4$: C 48.08, H 6.72, N 14.02, S 21.39%; found: C 47.70, H 6.48, N 13.98, S 21.64%; HRMS (ESI$^-$): m/z = 467.9957 {(TEA)[Ni(mnt)$_2$]}$^-$ (calcd = 467.9960), 337.8360 [Ni(mnt)$_2$]$^-$ (calcd = 337.8365). λ_{max} / cm (ε / L mol^{-1} cm^{-1}) = 215.5 (22980), 270.5 (43074), 316.0 (27320), 380.5 (6483), 473.5 (3567).

(TEA)$_2$[Cu(mnt)$_2$] = (TEA)$_2$Cu$_{mnt}$. The synthesis is adapted from that previously described with modifications.[6] To a yellow solution of Na_2mnt·1¾H_2O (686 mg, 3.15 mmol) in 20 mL 1:1 H_2O/EtOH was added a solution of $CuCl_2$ (212 mg, 1.58 mmol) in 2 mL H_2O resulting immediately in a dark brown solution. The reaction was stirred for 30 min, filtered through celite and washed with 10 mL 1:1 H_2O/EtOH. To the filtrate was added a 4 mL EtOH solution of TEACl (522 mg, 3.15 mmol) dropwise to give a maroon precipitate. After stirring for 45 min the maroon powder was filtered, washed with 5

mL water then 3 mL ice cold 1:1 H_2O/EtOH, and dried in air. The powder was recrystallized by dissolving in 15 mL hot acetone, adding 20 mL hot isopropanol then reducing the volume to 20 mL by heating, and cooling to 0°C. The resulting maroon microcrystalline solid was filtered, washed with 20 mL ice cold isopropanol, and dried in air then in vacuo to yield **$(TEA)_2[Cu(mnt)_2]$** (425 mg, 0.70 mmol, 45%). Anal calcd. for $C_{24}H_{40}CuN_6S_4$: C 47.69, H 6.67, N 13.90, S 21.22%; found: C 47.38, H 6.68, N 13.97, S 21.61%; HRMS (ESI$^-$): m/z = 472.9913 $\{(TEA)[Cu(mnt)_2]\}^-$ (calcd = 472.9903), 342.8304 $[Cu(mnt)_2]^-$ (calcd = 342.8307). λ_{max} / cm (ε / L mol^{-1} cm^{-1}) = 281.0 (22442), 319.5 (14976), 350.0 (10051), 370.0 (10153), 477.0 (4131).

Cyclic Voltammetry

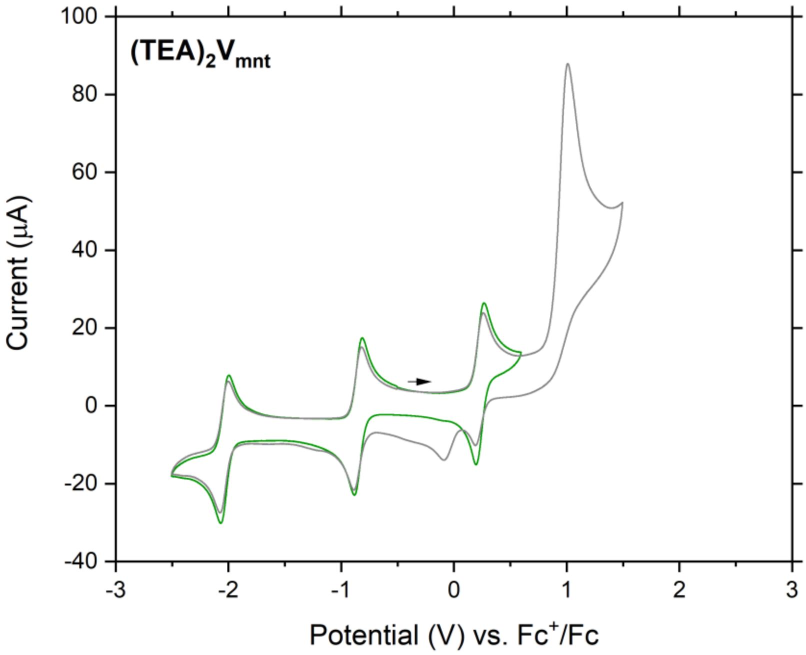

Figure S1. Second scan cyclic voltammograms of 1 mM $(TEA)_2$**V_{mnt}** in 0.1 M $TBAPF_6$ MeCN solution on glassy carbon electrode at 100 mV s^{-1}.

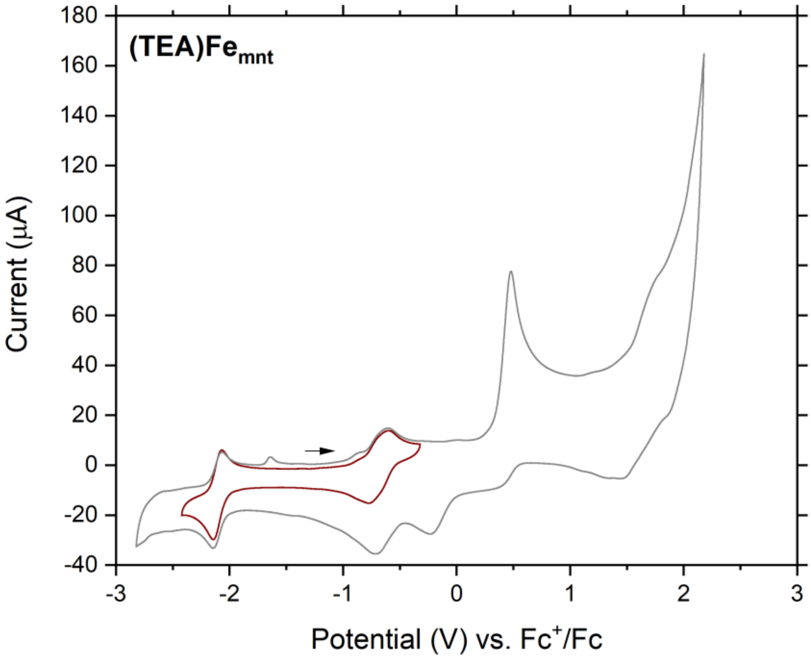

Figure S2. Second scan cyclic voltammograms of 1 mM (TEA)**Fe_{mnt}** in 0.1 M $TBAPF_6$ MeCN solution on glassy carbon electrode at 100 mV s^{-1}.

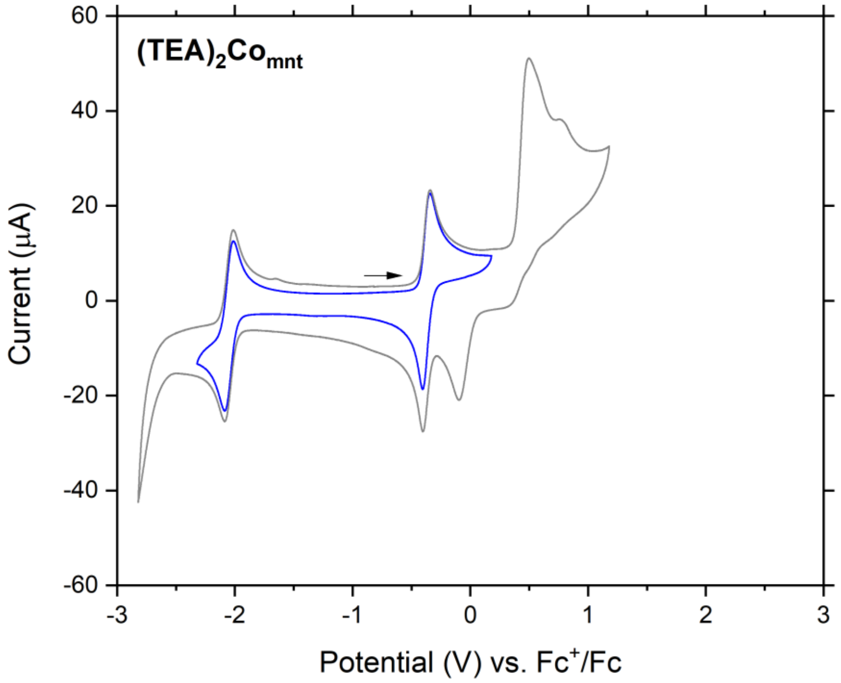

Figure S3. Second scan cyclic voltammograms of 1 mM $(TEA)_2$**Co_{mnt}** in 0.1 M $TBAPF_6$ MeCN solution on glassy carbon electrode at 100 mV s^{-1}.

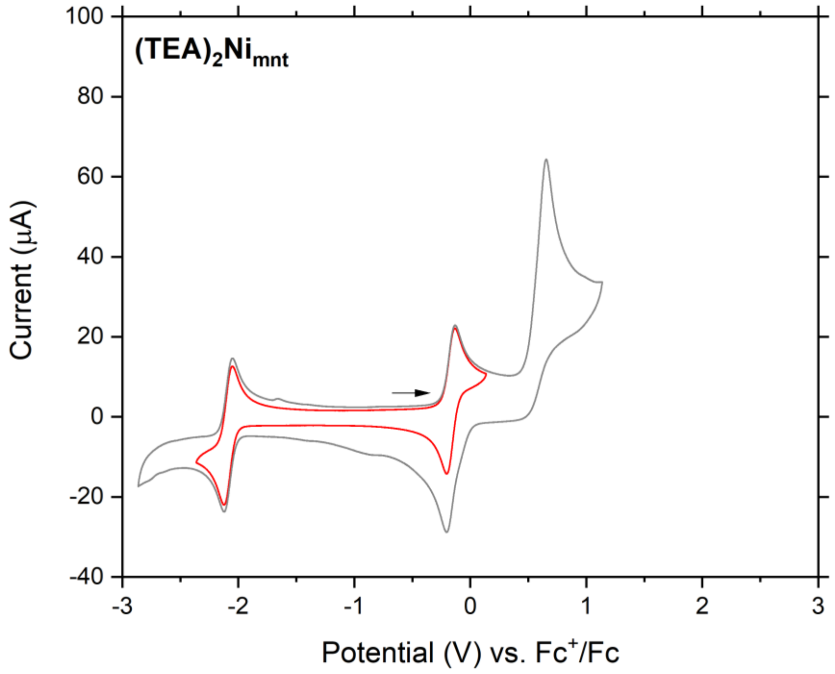

Figure S4. Second scan cyclic voltammograms of 1 mM $(TEA)_2$**Ni_{mnt}** in 0.1 M $TBAPF_6$ MeCN solution on glassy carbon electrode at 100 mV s^{-1}.

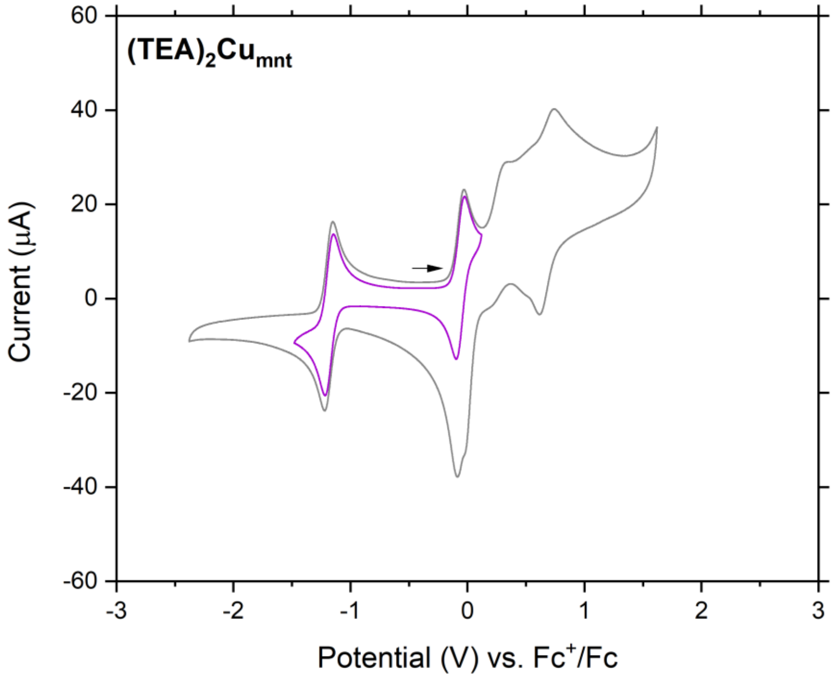

Figure S5. Second scan cyclic voltammograms of 1 mM $(TEA)_2$**Cu$_{mnt}$** in 0.1 M $TBAPF_6$ MeCN solution on glassy carbon electrode at 100 mV s^{-1}.

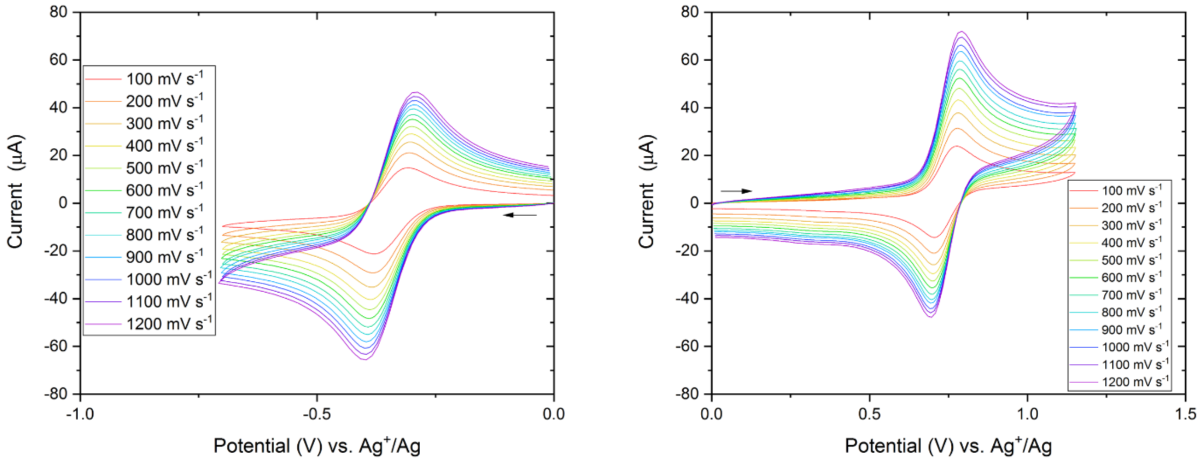

Figure S6. First scan cyclic voltammograms of the first reduction wave (left) and first oxidation wave (right) of 1 mM $(TEA)_2$**V$_{mnt}$** in 0.1 M $TBAPF_6$ MeCN solution on glassy carbon electrode at variable scan rates.

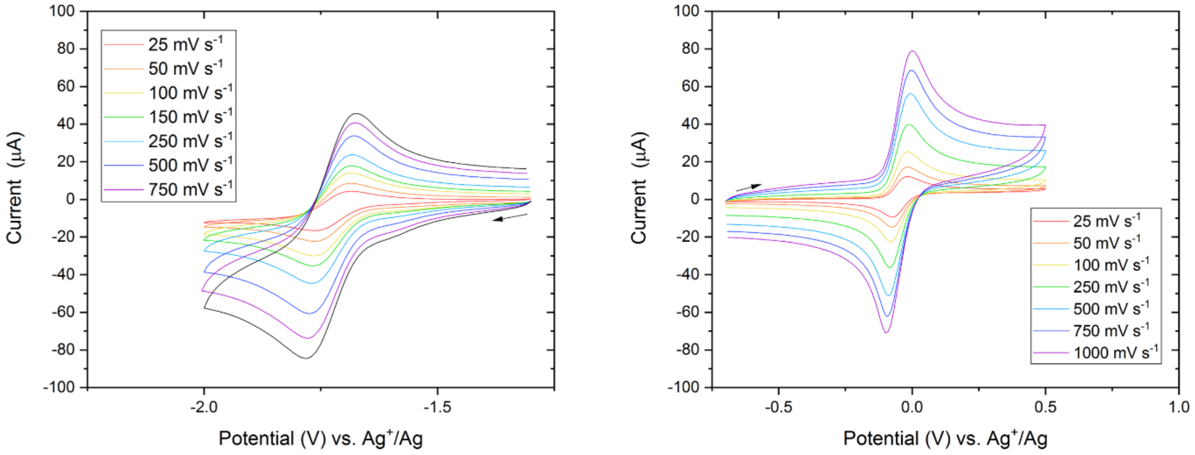

Figure S7. First scan cyclic voltammograms of the first reduction wave (left) and first oxidation wave (right) of 1 mM $(TEA)_2$**Co**$_{mnt}$ in 0.1 M $TBAPF_6$ MeCN solution on glassy carbon electrode at variable scan rates.

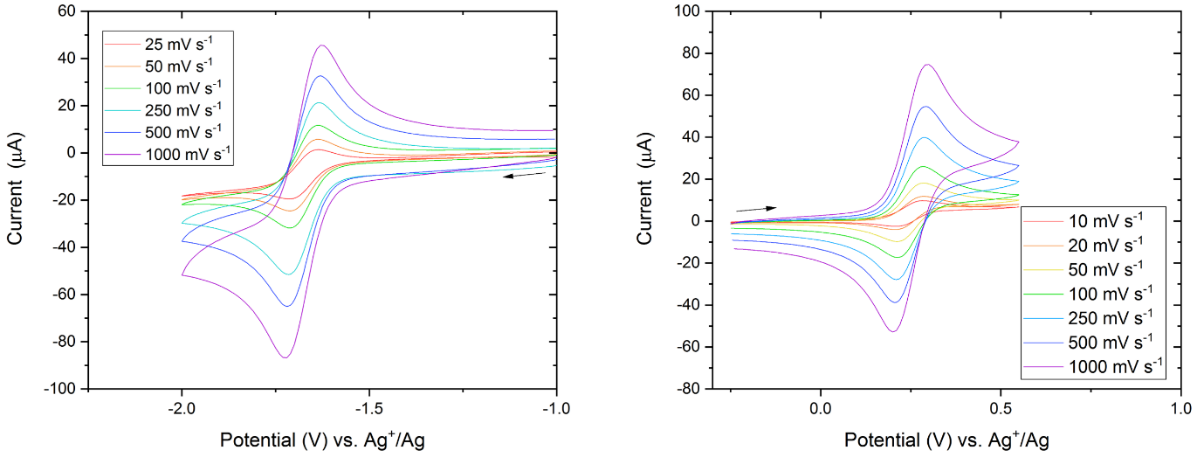

Figure S8. First scan cyclic voltammograms of the first reduction wave (left) and first oxidation wave (right) of 1 mM $(TEA)_2$**Ni**$_{mnt}$ in 0.1 M $TBAPF_6$ MeCN solution on glassy carbon electrode at variable scan rates.

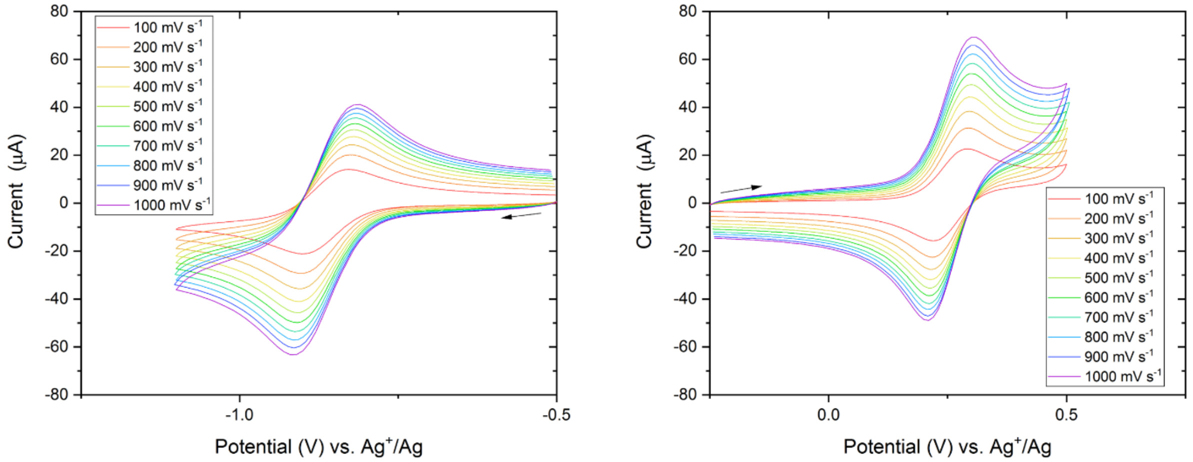

Figure S9. First scan cyclic voltammograms of the first reduction wave (left) and first oxidation wave (right) of 1 mM $(TEA)_2$**Cu_{mnt}** in 0.1 M $TBAPF_6$ MeCN solution on glassy carbon electrode at variable scan rates.

Table S1. Cell potentials [$E_{1/2}$(ox) – $E_{1/2}$(red)], solubility in acetonitrile, energy density of the five complexes, as well as the $E_{1/2}$, ΔE, diffusion coefficient and electrochemical rate constant for each of the two redox processes defining the cell potential.

**Complex**	**Cell potential**	**Solubility (mol L^{-1})**	**Energy density[a] (W h L^{-1})**	**$E_{1/2}$ vs. Fc^+/Fc[b][c] (V)**	**ΔE[b][c] (mV)**	**Diffusion coefficient[c] (cm^2 s^{-1})**	**Rate constant[c] (cm s^{-1})**
$(TEA)_2$**V**$_{mnt}$	1.08 V	0.53	7.7	-0.849	69	8.6×10^{-6} [d]	1.53×10^{-2}
				0.230	73	8.8×10^{-6} [e]	1.66×10^{-2}
$(TEA)_2$**V**$_{mnt}$	2.26 V	0.53	16	-2.032	74	-	-
				0.230	73	8.8×10^{-6} [e]	1.66×10^{-2}
(TEA)**Fe**$_{mnt}$	1.42 V	0.03	0.6	-2.106	77	-	-
				-0.689	177	-	-
$(TEA)_2$**Co**$_{mnt}$	1.68 V	0.39	8.8	-2.050	74	1.1×10^{-5} [f]	1.35×10^{-2}
				-0.375	61	1.3×10^{-5} [g]	1.16×10^{-2}
$(TEA)_2$**Ni**$_{mnt}$	1.92 V	0.30	7.7	-2.088	69	1.3×10^{-5} [h]	1.00×10^{-2}
				-0.166	70	1.4×10^{-5} [i]	1.63×10^{-2}
$(TEA)_2$**Cu**$_{mnt}$	1.12 V	0.91	14	-1.180	71	9.0×10^{-6} [j]	1.45×10^{-2}
				-0.061	69	9.3×10^{-6} [j]	1.39×10^{-2}

[a] Determined for a one electron transfer at the cell potential operating at maximum concentration. [b] Determined at 100 mV s^{-1} scan rate. [c] Conditions: 1 mM complex and 0.1 M $TBAPF_6$ in MeCN, glassy carbon working electrode, Pt wire counter electrode, Ag/Ag^+ quasi-reference electrode, N_2. [d] Determined for scan rates 100-600 mV s^{-1}. [e] Determined for scan rates 100-700 mV s^{-1}. [f] Determined for scan rates 25-500 mV s^{-1}. [g] Determined for scan rates 25-750 mV s^{-1}. [h] Determined for scan rates 25-500 mV s^{-1}. [i] Determined for scan rates 10-500 mV s^{-1}. [j] Determined for scan rates 50-500 mV s^{-1}.

Flow Battery Charge/Discharge Data

Galvanostatic battery experiments were performed using a conventional zero-gap flow-cell manufactured in house; the 'Gen 2 flow-cell' (Figures S10 and S11) was reproduced from literature.[7-8] The bodies of the flow-cell ('electrolyte diffusers') were CNC milled out of polypropylene, whereas the current collectors (flow-fields) were machined out of ppg86 (a graphite-polypropylene composite, Eisenhuth). Ethylene-propylene diene monomer (EPDM rubber, RH Nuttall, 0.5 mm) and non-compressible Polytetrafluoroethylene (PTFE, BOLA, 0.25 mm) were used as gasket and O-rings in order to seal the cell. The cell was assembled in the order of; current collector - carbon paper electrode - EPDM gasket - PTFE gasket - membrane - PTFE gasket - EPDM gasket - carbon paper electrode - current collector. Rubber gaskets were used to prevent leakage from the electrode compartment whereas PTFE gaskets were used to ensure that no electrical short-circuit occurred between the half-cells upon gasket compression. Before use, all plasticisers were removed from the EPDM rubber by storing the rubbers in a solvent mix (MeCN, acetone, methanol, isopropanol, ethanol and water) for at least 24h. The flow-cell was connected to perflouroalkoxy alkane (PFA, Savillex, 10 mL volume) reservoirs via PFA tubing (1/8" OD). Norprene peristaltic pump tubing (Cole-Parmer, Masterflex L/S 14) was used to connect the flow-cell to a Masterflex L/S series peristaltic pump equiped with an Easy-Load II pumphead (Cole-Parmer). Stainless-steel reducing unions (Pipeline Products Direct) were used to unite the different tubings which each had an inner diameter of 1.6 mm. The flow-rate was prior calibrated against the pump rpm by use of pure MeCN.

For the attempted higher potential (2.26 V) flow battery of $(TEA)_2\mathbf{V}_{mnt}$ (Figure S14 and Figure S17) each reservoir was filled with $(TEA)_2\mathbf{V}_{mnt}$ electrolyte solution, and one charge cycle to 1.5 V was performed (cycle 0) and the electrolytes returned to the reservoirs. The posolyte reservoir was drained and replaced with fresh $(TEA)_2\mathbf{V}_{mnt}$ electrolyte, such that the posolyte was $\mathbf{V}_{mnt}^{1-}$ and the negolyte was $\mathbf{V}_{mnt}^{3-}$, then the battery cycling with charging up to 2.7 V commenced.

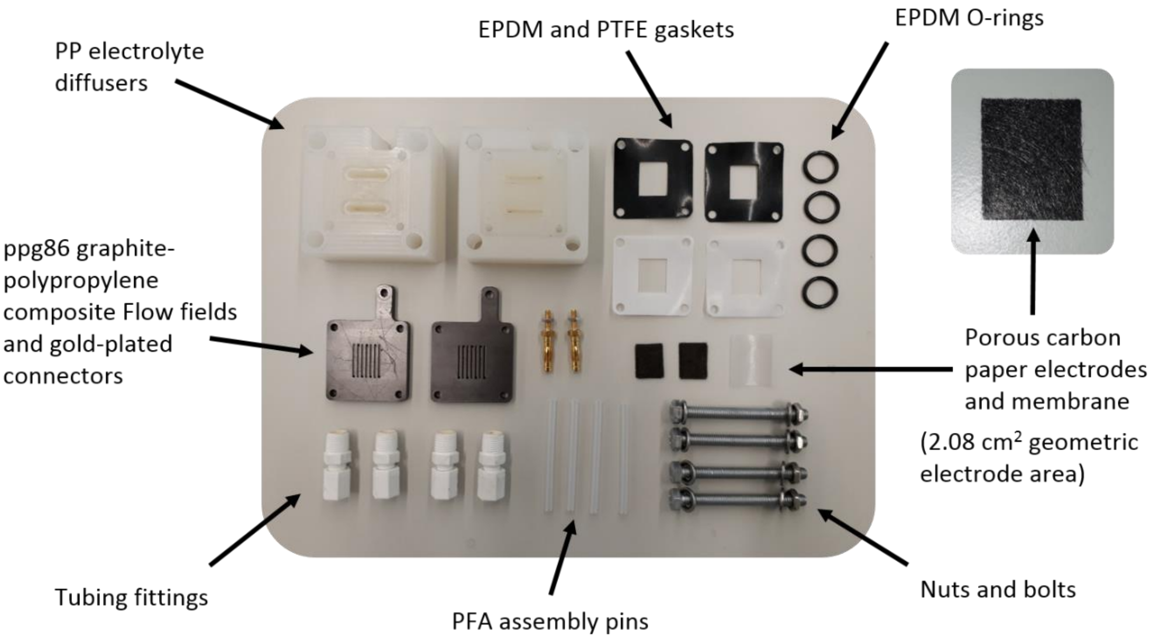

Figure S10. Disassembled non-aqueous flow-cell with a 2.08 cm^2 electrode area.

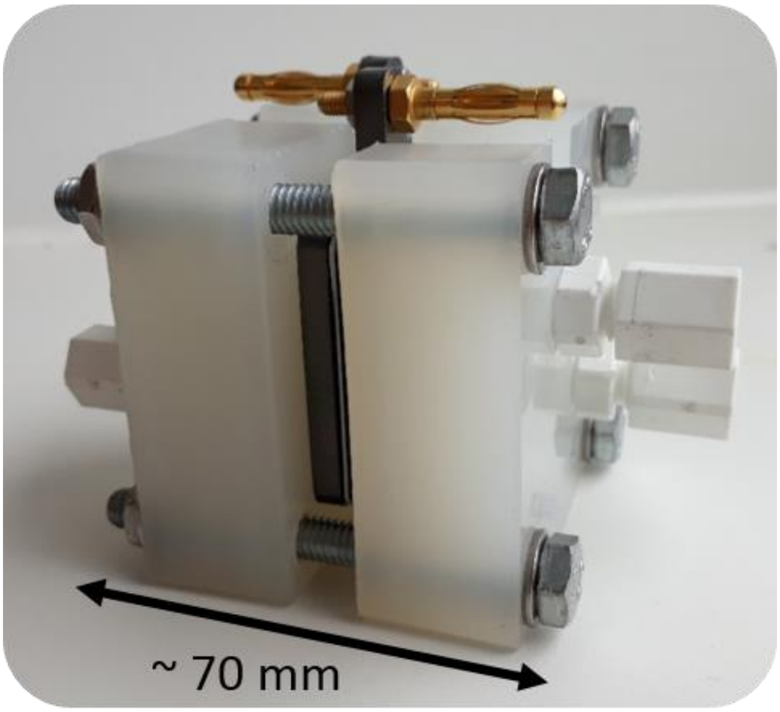

Figure S11. Fully assembled Gen-2 flow-cell.

$(TEA)_2V_{mnt}$ in MeCN, fumapem F-930 cation exchange membrane separator.

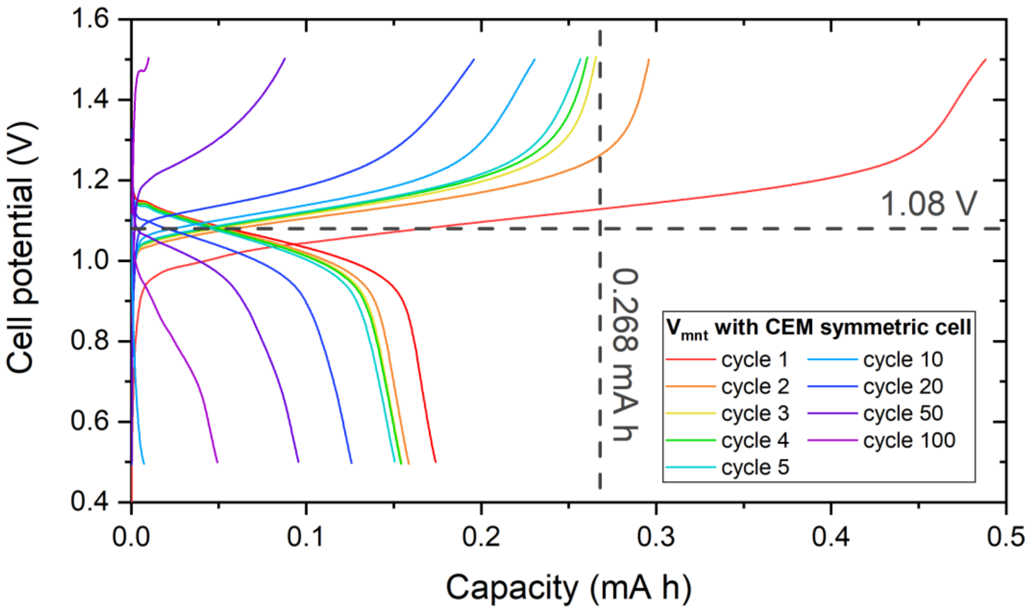

Figure S12. Cell potential vs. capacity for selected charge/discharge cycles at ±0.48 mA cm^{-2} for 1 mM $(TEA)_2$**V_{mnt}** in 0.1 M $TBAPF_6$ MeCN solution with fumapem F-930 cation exchange membrane. Dashed grey lines indicate the theoretical cell potential and capacity.

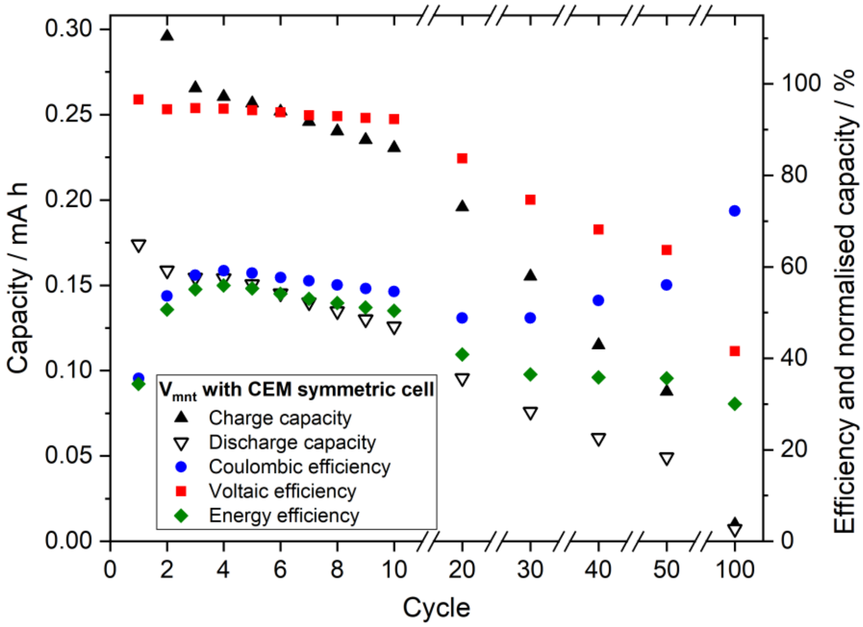

Figure S13. Efficiencies and charge/discharge capacities for selected cycles at ±0.48 mA cm^{-2} for 1 mM $(TEA)_2$**V_{mnt}** in 0.1 M $TBAPF_6$ MeCN solution with fumapem F-930 cation exchange membrane.

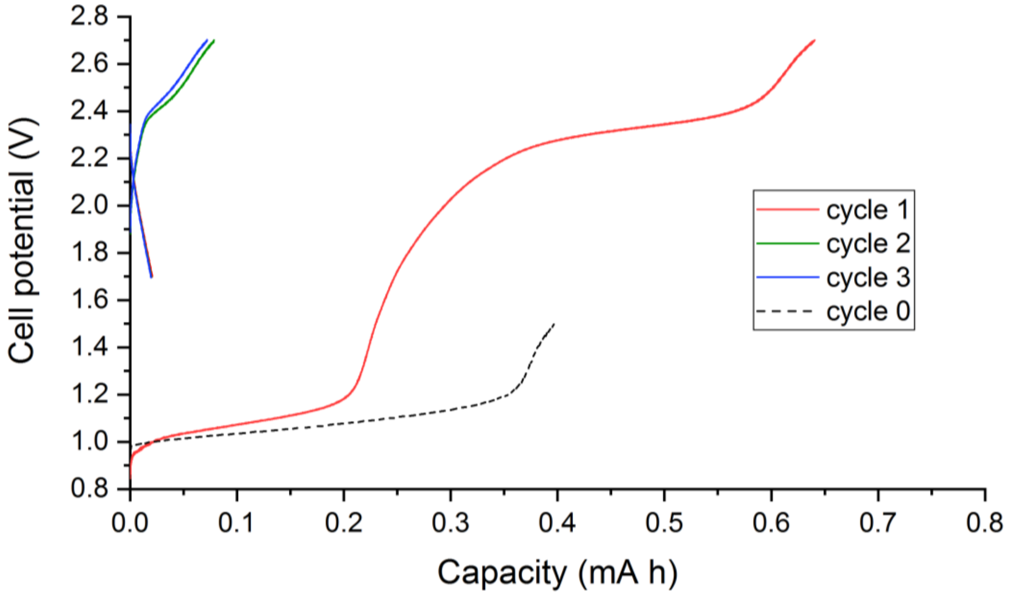

Figure S14. Cell potential vs. capacity for the first three charge/discharge cycles at ±1 mA for the 2.26 V potential battery of 1 mM $(TEA)_2\mathbf{V_{mnt}}$ in 0.1 M $TBAPF_6$ MeCN solution with fumapem F-930 cation exchange membrane separator. Also shown is 'cycle 0', the initial charge cycle to 1.5 V before the posolyte was replaced with fresh $(TEA)_2\mathbf{V_{mnt}}$ solution.

$(TEA)_2V_{mnt}$ in propylene carbonate, Celgard separator.

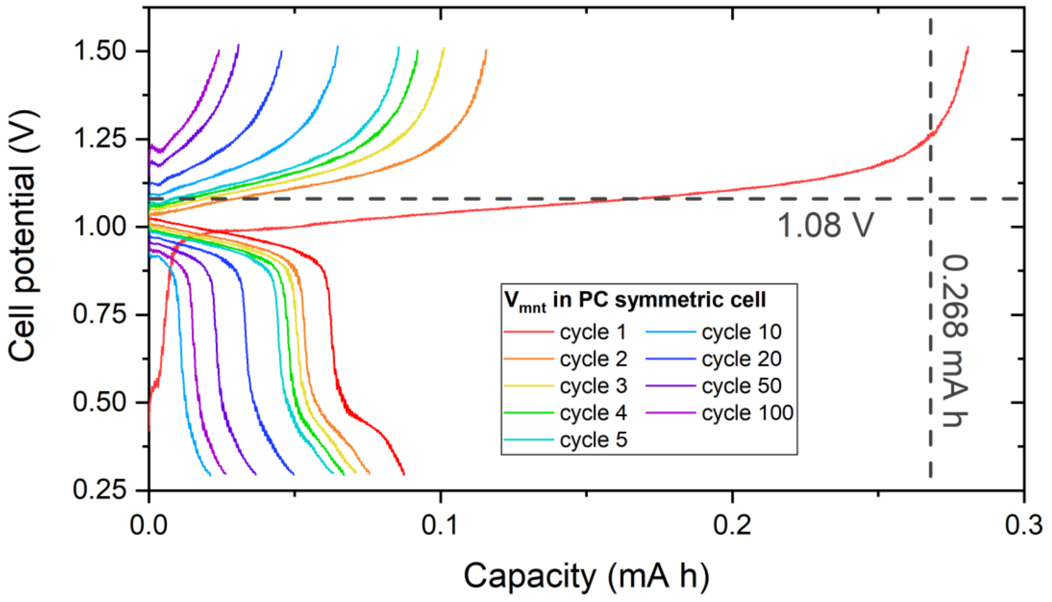

Figure S15. Cell potential vs. capacity for selected charge/discharge cycles at ±0.24 mA cm^{-2} for 1 mM $(TEA)_2\mathbf{V_{mnt}}$ in 0.1 M $TBAPF_6$ propylene carbonate solution with Celgard separator. Dashed grey lines indicate the theoretical cell potential and capacity.

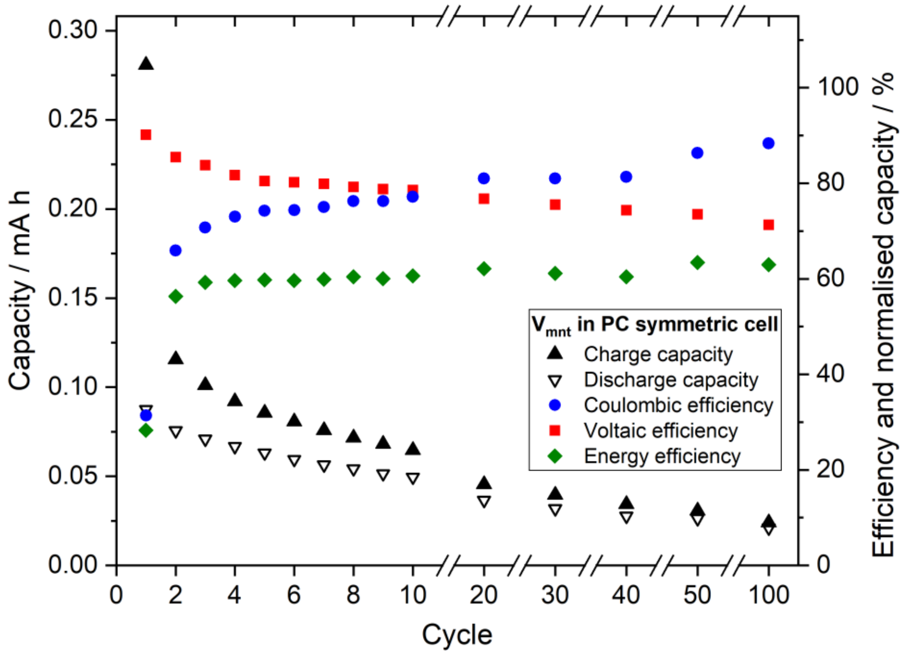

Figure S16. Efficiencies and charge/discharge capacities for selected cycles at ±0.48 mA cm^{-2} for 1 mM $(TEA)_2$**V$_{mnt}$** in 0.1 M $TBAPF_6$ propylene carbonate solution with Celgard separator.

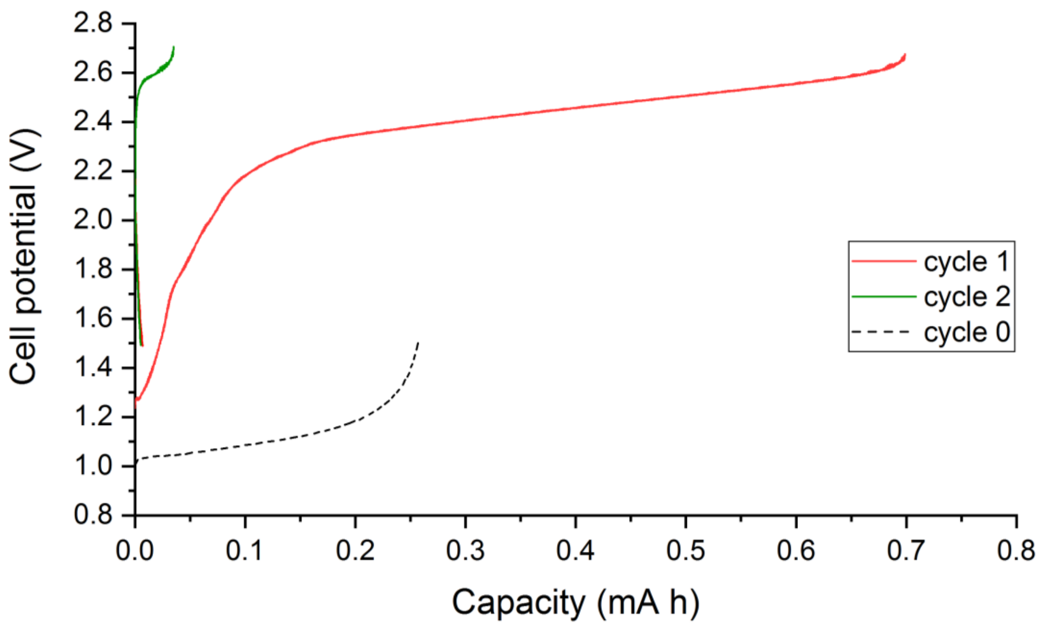

Figure S17. Cell potential vs. capacity for the first two charge/discharge cycles at ±0.24 mA cm^{-2} for the 2.26 V potential battery of 1 mM $(TEA)_2$**V$_{mnt}$** in 0.1 M $TBAPF_6$ propylene carbonate solution with Celgard separator. Also shown is 'cycle 0', the initial charge cycle to 1.5 V before the posolyte was replaced with fresh $(TEA)_2$**V$_{mnt}$** solution.

(TEA)Fe$_{mnt}$ in MeCN, Celgard separator.

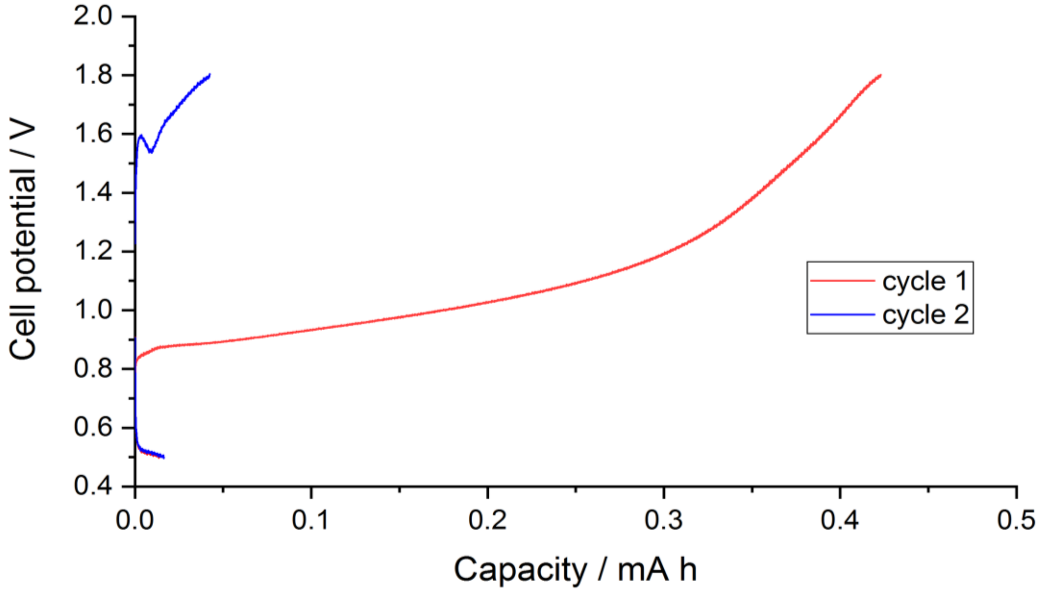

Figure S18. Cell potential vs. capacity for the first two charge/discharge cycles at ±0.48 mA cm^{-2} for 1 mM (TEA)**Fe$_{mnt}$** in 0.1 M TBAPF$_6$ MeCN solution with Celgard separator.

(TEA)$_2$Ni$_{mnt}$ in MeCN, Celgard separator.

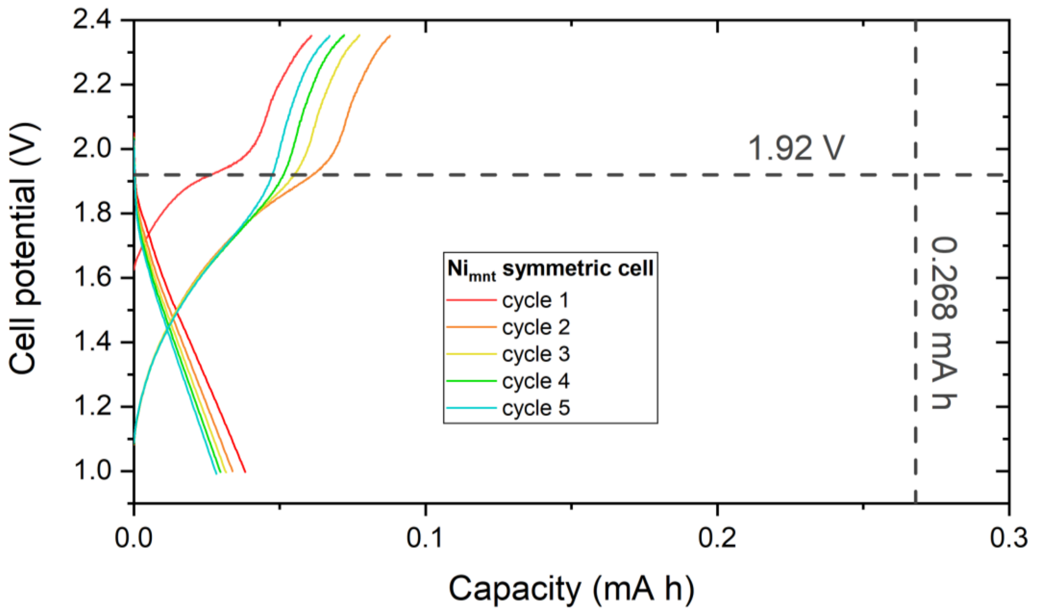

Figure S19. Cell potential vs. capacity for the first five charge/discharge cycles at ±0.24 mA cm^{-2} for 1 mM (TEA)$_2$**Ni$_{mnt}$** in 0.1 M TBAPF$_6$ MeCN solution with Celgard separator. Dashed grey lines indicate the theoretical cell potential and capacity.

$(TEA)_2$Ni$_{mnt}$ in propylene carbonate, Celgard separator.

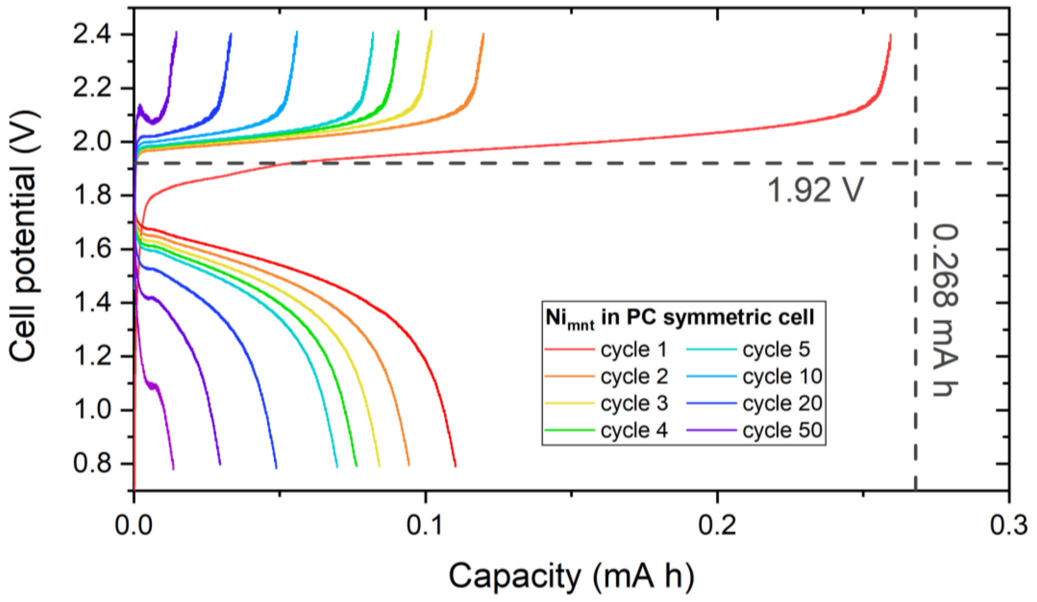

Figure S20. Cell potential vs. capacity for selected charge/discharge cycles at ±0.48 mA cm^{-2} for 1 mM $(TEA)_2$**Ni$_{mnt}$** in 0.1 M $TBAPF_6$ propylene carbonate solution with Celgard separator. Dashed grey lines indicate the theoretical cell potential and capacity.

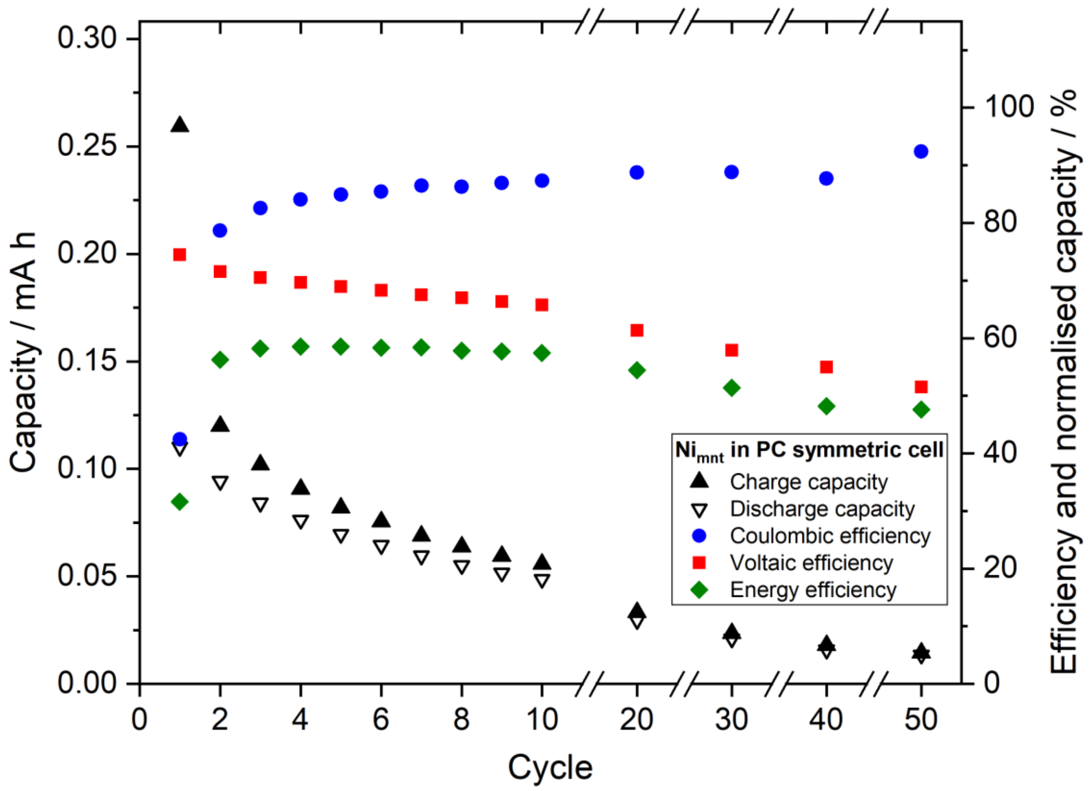

Figure S21. Efficiencies and charge/discharge capacities for selected cycles at ±0.48 mA cm^{-2} for 1 mM $(TEA)_2$**Ni$_{mnt}$** in 0.1 M $TBAPF_6$ propylene carbonate solution with Celgard separator.

$(TEA)_2Cu_{mnt}$ in MeCN, Celgard separator.

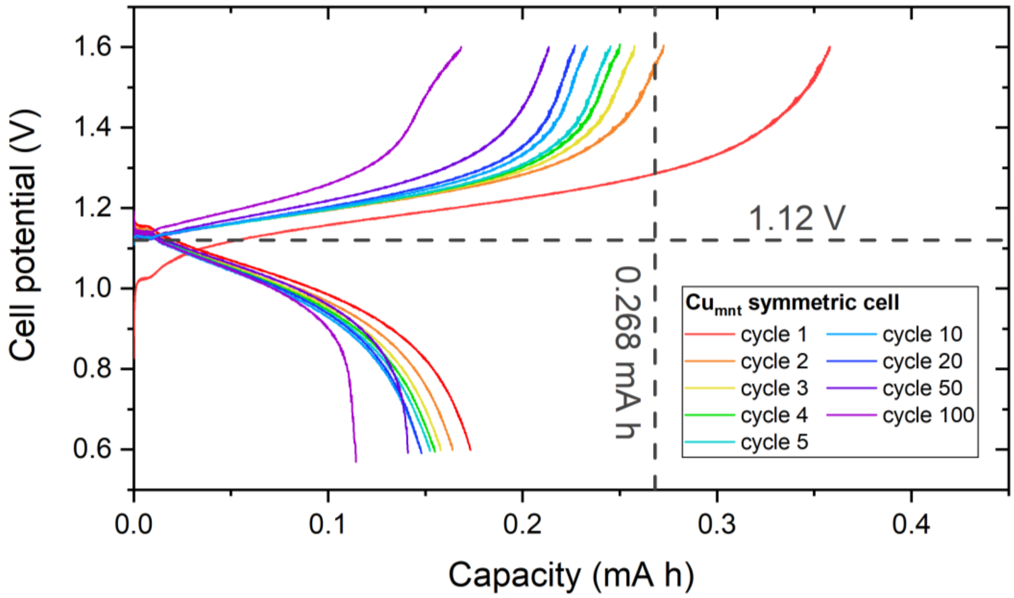

Figure S22. Cell potential vs. capacity for selected charge/discharge cycles at ±0.48 mA cm^{-2} for 1 mM $(TEA)_2$**Cu_{mnt}** in 0.1 M $TBAPF_6$ MeCN solution with Celgard separator.

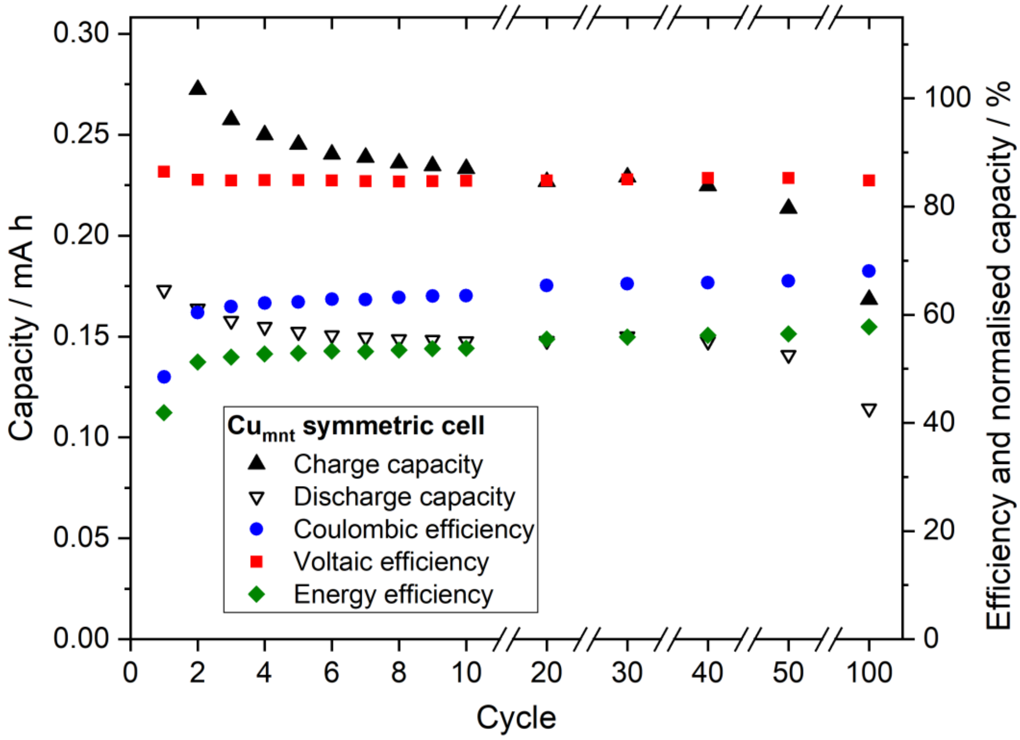

Figure S23. Efficiencies and charge/discharge capacities for selected cycles at ±0.48 mA cm^{-2} for 1 mM $(TEA)_2$**Cu_{mnt}** in 0.1 M $TBAPF_6$ MeCN solution with Celgard separator.

(TEA)$_2$Cu$_{mnt}$ in MeCN, fumapem F-930 cation exchange membrane separator.

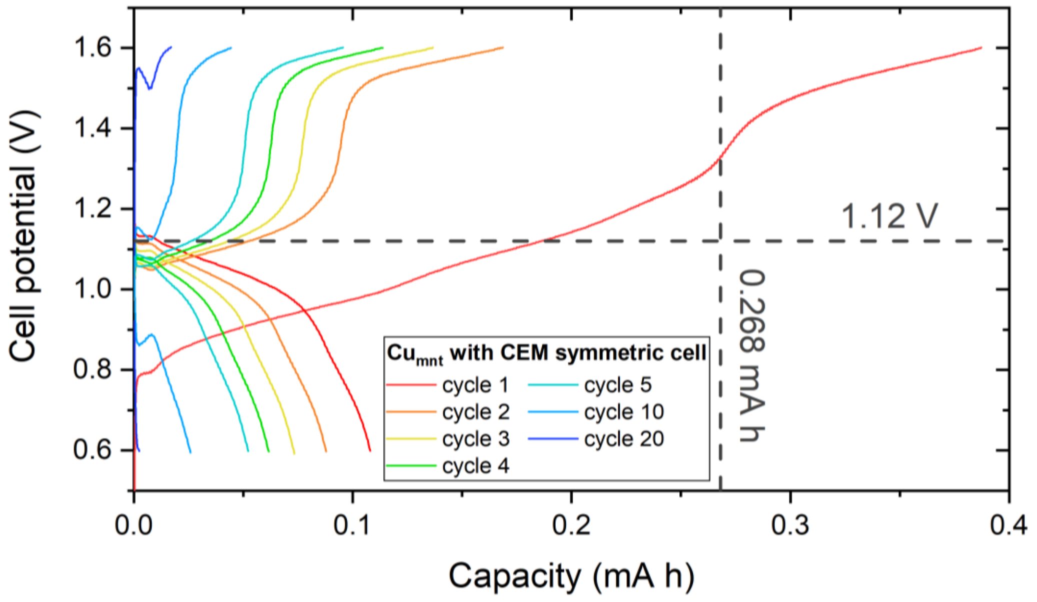

Figure S24. Cell potential vs. capacity for selected charge/discharge cycles at ±0.48 mA cm^{-2} for 1 mM (TEA)$_2$**Cu$_{mnt}$** in 0.1 M TBAPF$_6$ MeCN solution with fumapem F-930 cation exchange membrane separator.

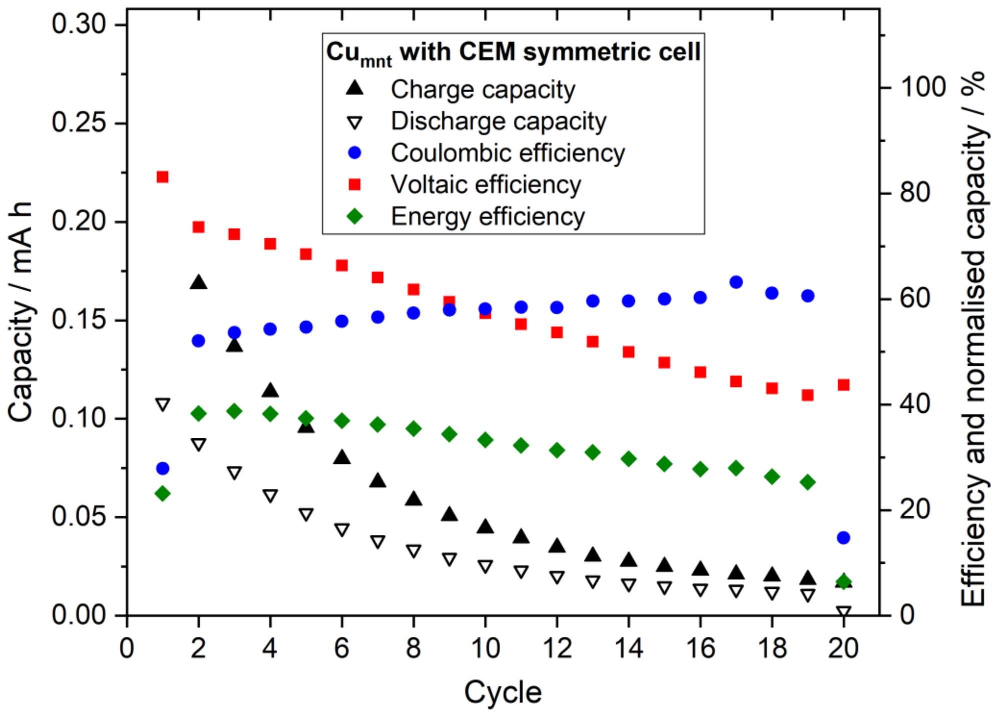

Figure S25. Efficiencies and capacities for the first 20 charge/discharge cycles at ±0.48 mA cm^{-2} for 1 mM (TEA)$_2$**Cu$_{mnt}$** in 0.1 M TBAPF$_6$ MeCN solution with fumapem F-930 cation exchange membrane separator.

Table S2. Coulombic, voltaic, and energy efficiencies as well as discharge capacities for the 2nd, 5th, 10th, and 50th cycles of the flow cell experiments.

	**Coulombic efficiency (%)**				**voltaic efficiency (%)**				**energy efficiency (%)**				**discharge capacity (mA h)**			
**cycle no. →** / **complex/current/ solvent/separator/↓**	**2**	**5**	**10**	**50**	**2**	**5**	**10**	**50**	**2**	**5**	**10**	**50**	**2**	**5**	**10**	**50**
$(TEA)_2$**V**$_{mnt}$/ MeCN/Celgard	62	62	62	71	95	95	96	59	59	59	60	42	0.158	0.158	0.150	0.053
$(TEA)_2$**V**$_{mnt}$ / MeCN/F-930	54	59	55	56	94	94	92	64	51	55	50	36	0.159	0.151	0.126	0.049
$(TEA)_2$**V**$_{mnt}$ / PC/Celgard	66	74	77	86	70	66	63	54	46	49	48	47	0.076	0.063	0.050	0.026
$(TEA)_2$**Co**$_{mnt}$ / MeCN/Celgard	63	66	69	72	96	96	96	80	61	64	66	58	0.133	0.113	0.099	0.037
$(TEA)_2$**Ni**$_{mnt}$ / PC/Celgard	79	85	87	92	72	69	66	52	56	59	57	48	0.094	0.070	0.049	0.013
$(TEA)_2$**Cu**$_{mnt}$ / MeCN/Celgard	60	62	63	66	85	85	85	85	51	53	54	57	0.169	0.152	0.147	0.141
$(TEA)_2$**Cu**$_{mnt}$ / MeCN/F-930[a]	52	55	58	-	74	68	57	-	38	37	33	-	0.088	0.052	0.026	-

[a] Experiment was aborted after 20 cycles as the capacity had faded to 0.002 mA h.

Independent single redox couple '0 V' battery cycling

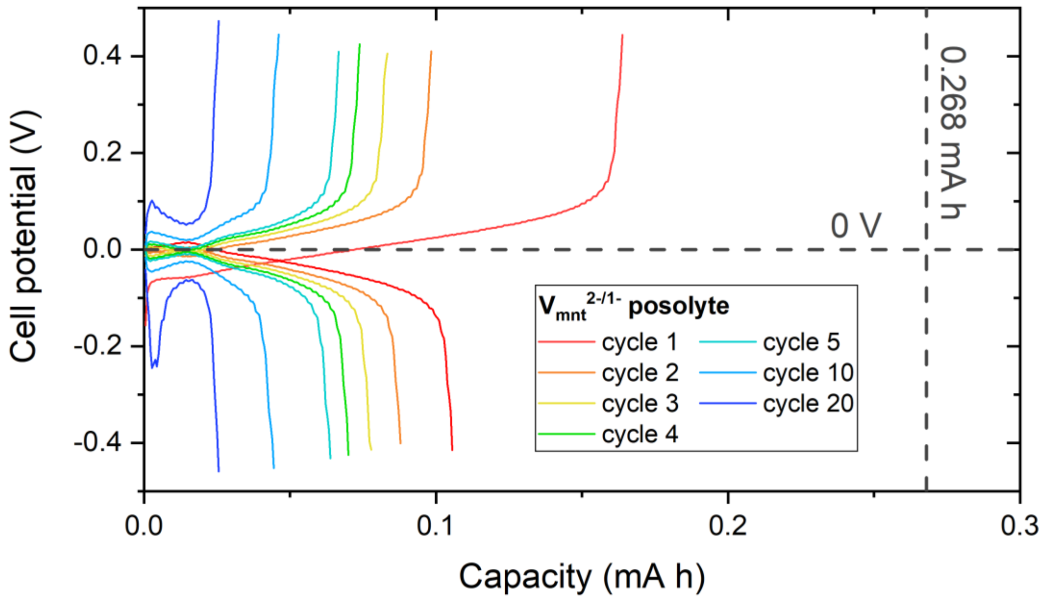

Figure S26. Cell potential vs. capacity for selected cycles of the $\mathbf{V_{mnt}}^{2-/1-}$ posolyte-only single redox couple cell. Conditions: 1mM $(TEA)_2$$\mathbf{V_{mnt}}$ in 0.1 M $TBAPF_6$ MeCN solution, 10 mL volume each half cell, 10 mL min^{-1} flow rate, ±0.96 mA cm^{-2} charge/discharge current, 0.4/-0.4 V charge/discharge potential thresholds, Celgard separator.

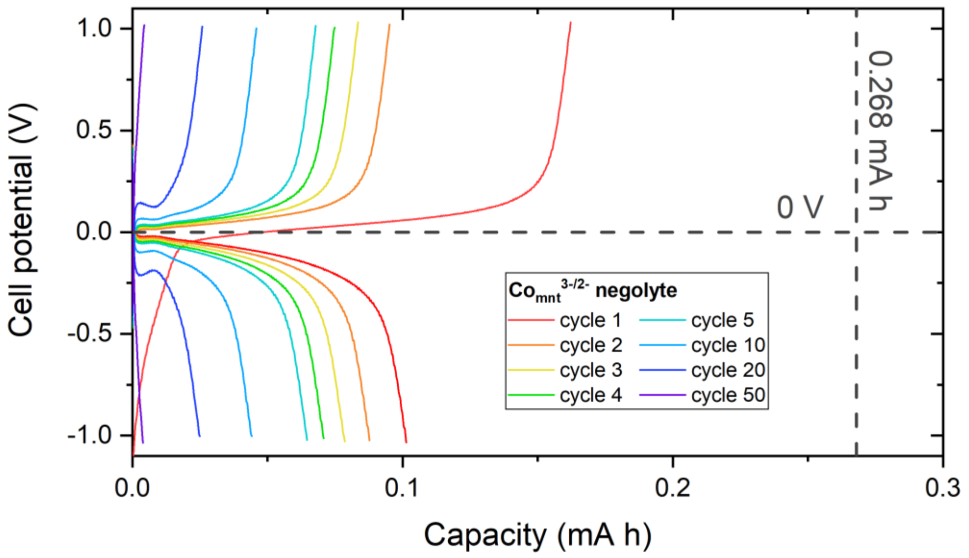

Figure S27. Cell potential vs. capacity for selected cycles of the $\mathbf{Co_{mnt}}^{3-/2-}$ negolyte-only single redox couple cell. Conditions: 1mM $(TEA)_2$$\mathbf{Co_{mnt}}$ in 0.1 M $TBAPF_6$ MeCN solution, 10 mL volume each half cell, 10 mL min^{-1} flow rate, ±0.48 mA cm^{-2} charge/discharge current, 1.0/-1.0 V charge/discharge potential thresholds, Celgard separator.

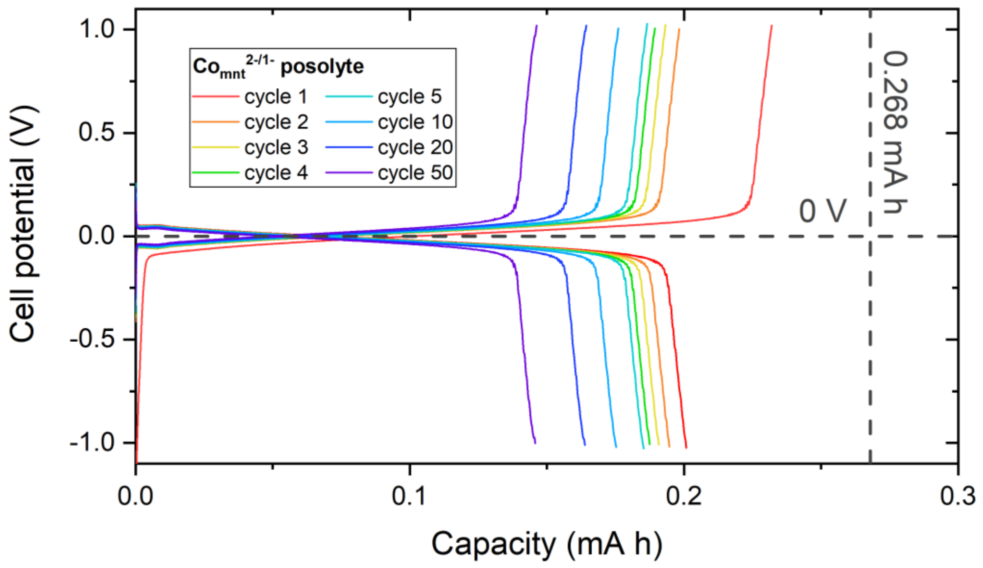

Figure S28. Cell potential vs. capacity for selected cycles of the **Co$_{mnt}$$^{2-/1-}$** posolyte-only single redox couple cell. Conditions: 1mM $(TEA)_2$**Co$_{mnt}$** in 0.1 M $TBAPF_6$ MeCN solution, 10 mL volume each half cell, 10 mL min^{-1} flow rate, ±0.48 mA cm^{-2} charge/discharge current, 1.0/-1.0 V charge/discharge potential thresholds, Celgard separator.

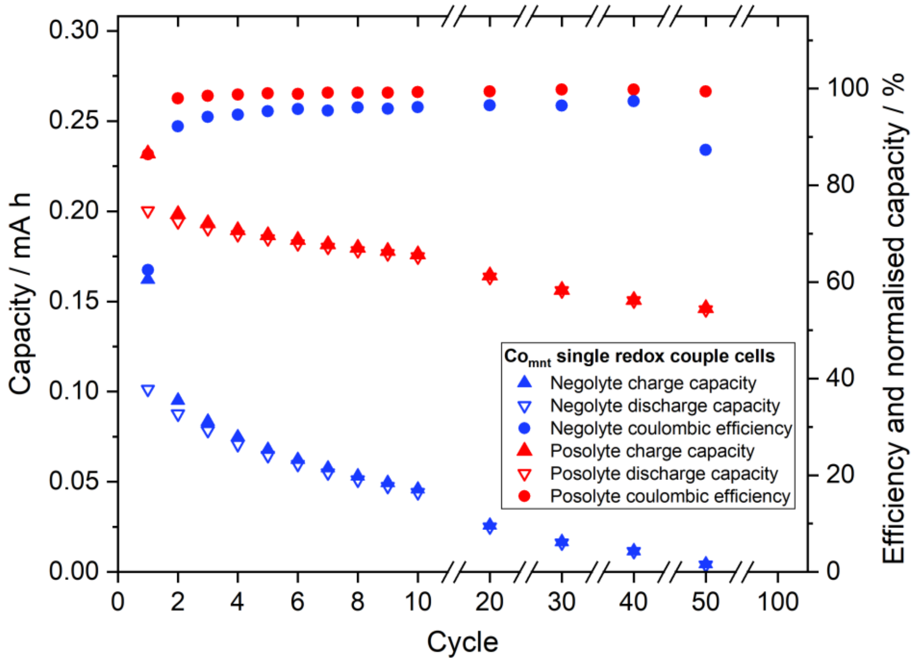

Figure S29. Charge/discharge capacities and coulombic efficiencies for selected cycles of the **Co$_{mnt}$$^{3-/2-}$** negolyte-only (blue data) and **Co$_{mnt}$$^{2-/1-}$** posolyte-only (red data) single redox couple cell. Conditions: 1mM $(TEA)_2$**Co$_{mnt}$** in 0.1 M $TBAPF_6$ MeCN solution, 10 mL volume each half cell, 10 mL min^{-1} flow rate,

±0.48 mA cm^{-2} charge/discharge current, 1.0/-1.0 V charge/discharge potential thresholds, Celgard separator.

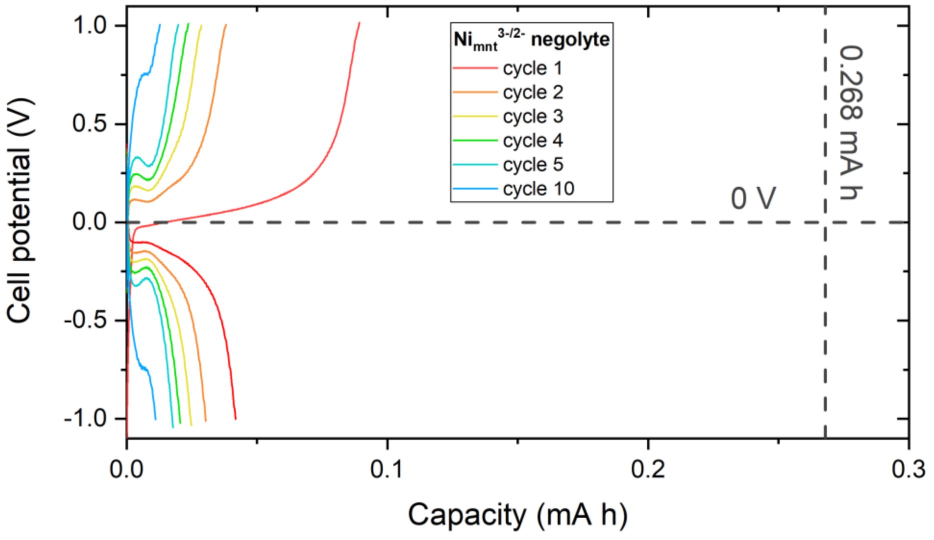

Figure S30. Cell potential vs. capacity for selected cycles of the **Ni**$_{\mathbf{mnt}}$$^{3-/2-}$ negolyte-only single redox couple cell. Conditions: 1mM $(TEA)_2$**Ni**$_{\mathbf{mnt}}$ in 0.1 M $TBAPF_6$ MeCN solution, 10 mL volume each half cell, 10 mL min^{-1} flow rate, ±0.48 mA cm^{-2} charge/discharge current, 1.0/-1.0 V charge/discharge potential thresholds, Celgard separator.

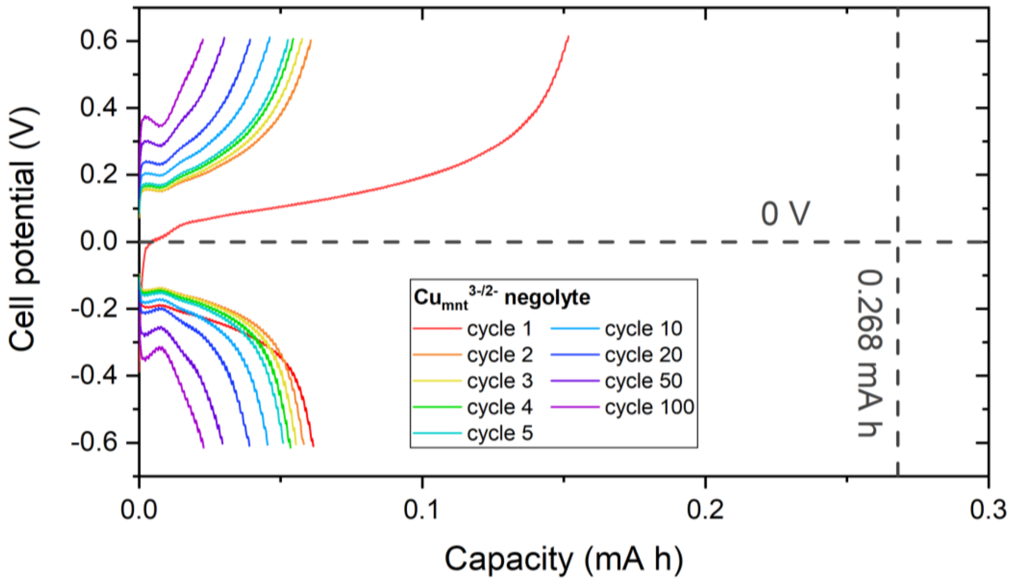

Figure S31. Cell potential for selected cycles of the **Cu**$_{\mathbf{mnt}}$$^{3-/2-}$ negolyte-only single redox couple cell. Conditions: 1mM $(TEA)_2$**Cu**$_{\mathbf{mnt}}$ in 0.1 M $TBAPF_6$ MeCN solution, 10 mL volume each half cell, 10 mL min^{-1} flow rate, ±0.48 mA cm^{-2} charge/discharge current, 0.6/-0.6 V charge/discharge potential thresholds, Celgard separator.

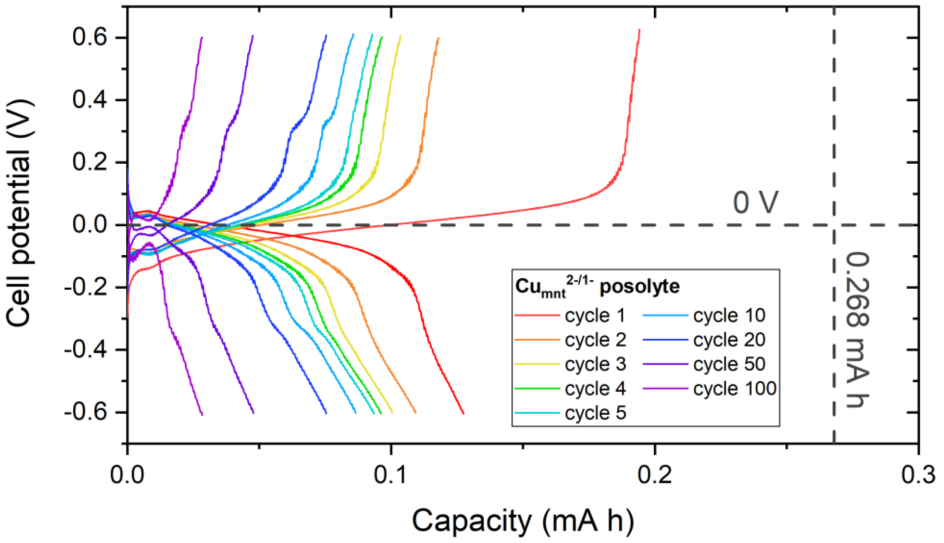

Figure S32. Cell potential vs. capacity for selected cycles of the **Cu$_{mnt}$**$^{2-/1-}$ posolyte-only single redox couple cell. Conditions: 1 mM $(TEA)_2$**Cu$_{mnt}$** in 0.1 M $TBAPF_6$ MeCN solution, 10 mL volume each half cell, 10 mL min^{-1} flow rate, ±0.48 mA cm^{-2} charge/discharge current, 0.6/-0.6 V charge/discharge potential thresholds, Celgard separator.

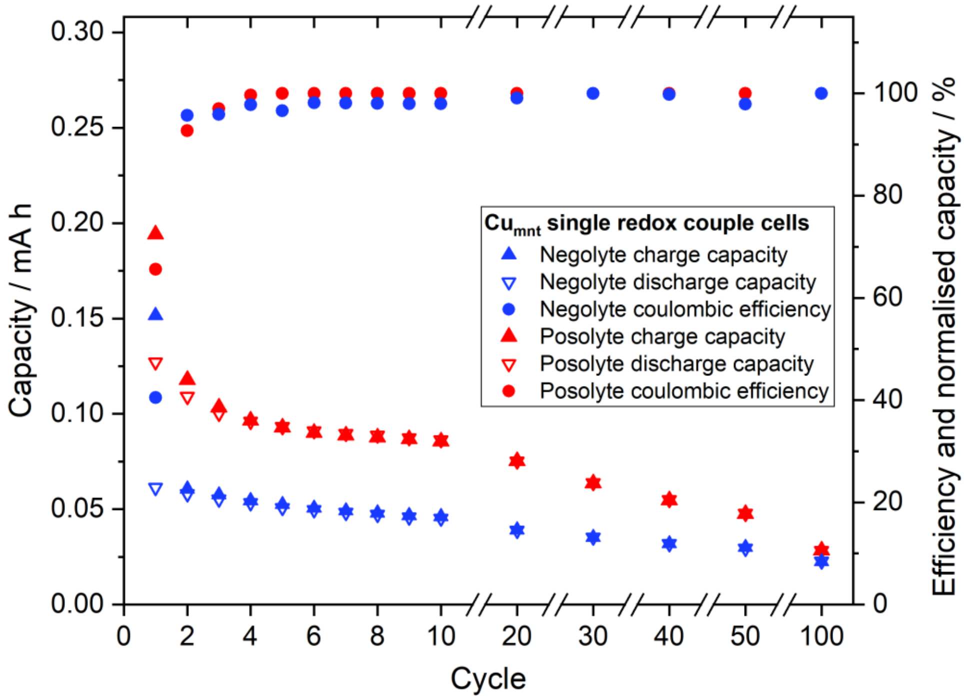

Figure S33. Charge/discharge capacities and coulombic efficiencies for selected cycles of the **Cu$_{mnt}$**$^{3-/2-}$ negolyte-only (blue data) and **Cu$_{mnt}$**$^{2-/1-}$ posolyte-only (red data) single redox couple cell. Conditions: 1 mM $(TEA)_2$**Cu$_{mnt}$** in 0.1 M $TBAPF_6$ MeCN solution, 10 mL volume each half cell, 10 mL min^{-1} flow rate, ±0.48 mA cm^{-2} charge/discharge current, 0.6/-0.6 V charge/discharge potential thresholds, Celgard separator.

UV-vis spectroscopy

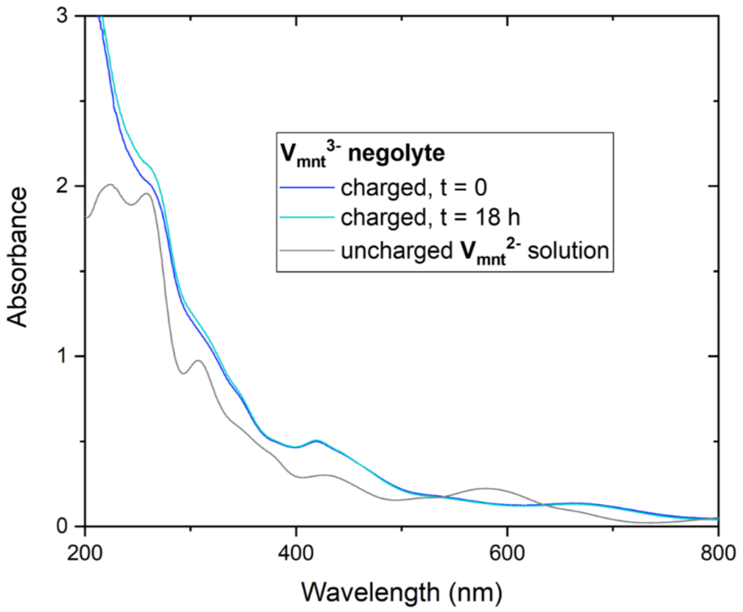

Figure S34. Time evolution of the UV-vis spectra of the $\mathbf{V_{mnt}}^{3-}$ negolyte solution solution diluted by a factor of 20 to 50 µM $\mathbf{V_{mnt}}^{n-}$ in 5 mM $TBAPF_6$ MeCN solution, of the 1.09 V battery of $(TEA)_2\mathbf{V_{mnt}}$ after an initial charge cycle to 1.5 V. Also shown is the uncharged starting solution (grey), 50 µM $(TEA)_2\mathbf{V_{mnt}}$ MeCN solution.

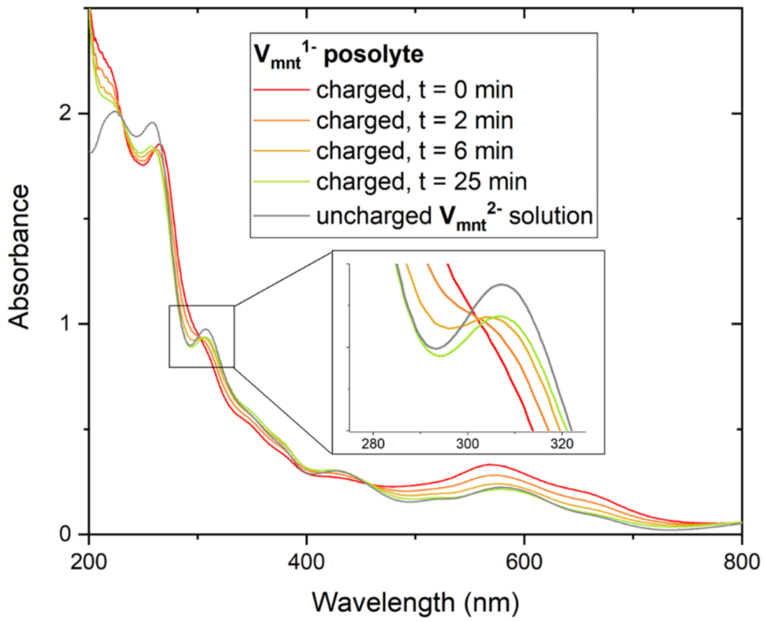

Figure S35. Time evolution of the UV-vis spectra of the $\mathbf{V_{mnt}}^{-}$ posolyte solution diluted by a factor of 20 to 50 µM $\mathbf{V_{mnt}}^{n-}$ in 5 mM $TBAPF_6$ MeCN solution, of the 1.09 V battery of $(TEA)_2\mathbf{V_{mnt}}$ after an initial charge cycle to 1.5 V. Also shown is the uncharged starting solution (grey), 50 µM $(TEA)_2\mathbf{V_{mnt}}$ MeCN solution.

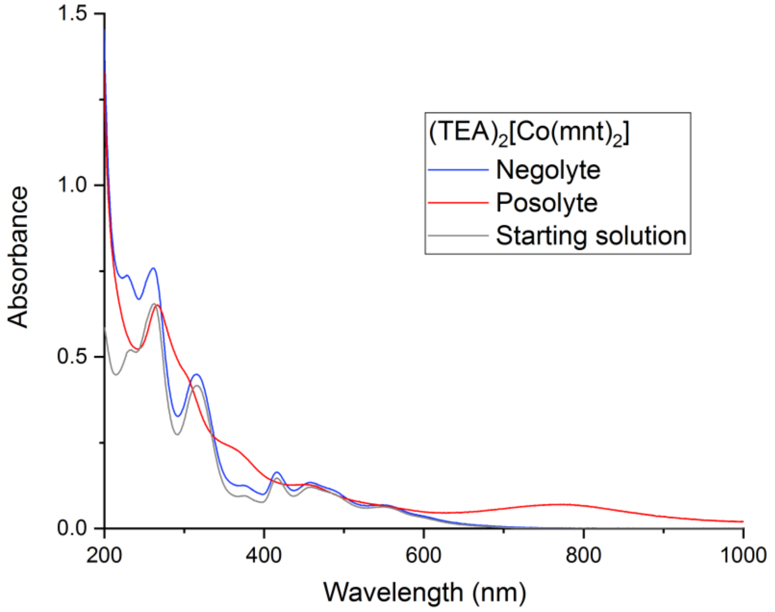

Figure S36. UV-vis spectra of the negolyte (blue) and posolyte (red) solutions, diluted by a factor of 20 to 50 µM **Co$_{mnt}$**$^{n-}$ in 5 mM TBAPF$_6$ MeCN solution, after an initial charge cycle to 1.2 V. Also shown is the uncharged starting solution of each half cell (grey), (TEA)$_2$**Co$_{mnt}$** diluted by a factor of 20 to 50 µM in 5 mM TBAPF$_6$ MeCN solution.

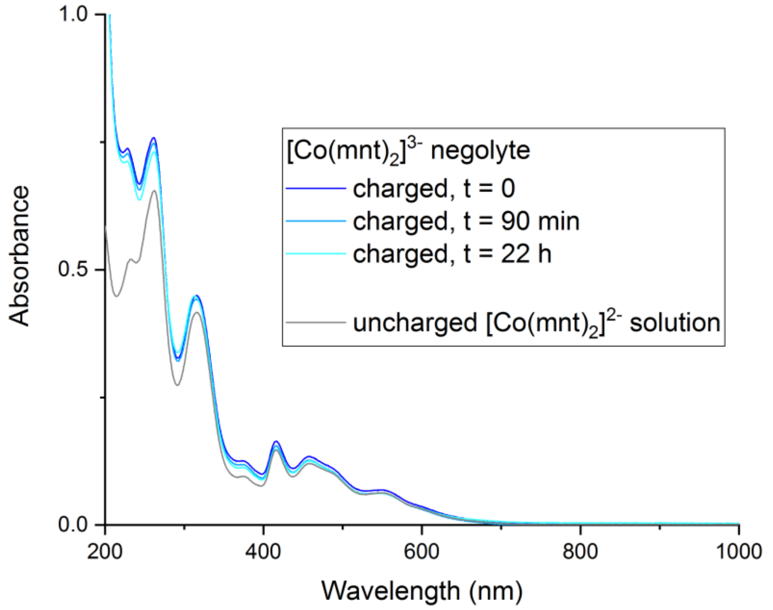

Figure S37. Time evolution of the UV-vis spectra of the negolyte solution of (TEA)$_2$**Co$_{mnt}$** (dark to light blue with time), diluted by a factor of 20 to 50 µM **Co$_{mnt}$**$^{n-}$ in 5 mM TBAPF$_6$ MeCN solution, after an initial charge cycle to 2.2 V. Also shown is the uncharged starting solution (grey), (TEA)$_2$**Co$_{mnt}$** diluted by a factor of 20 to 50 µM in 5 mM TBAPF$_6$ MeCN solution.

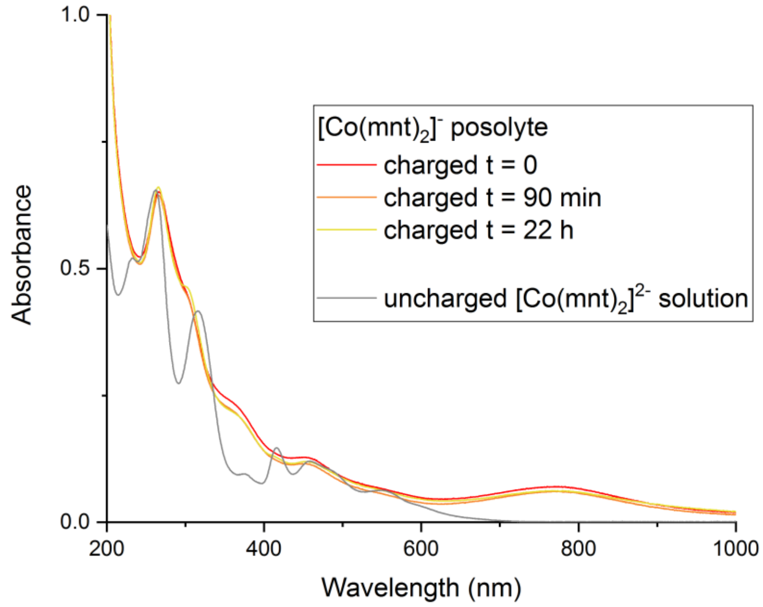

Figure S38. Time evolution of the UV-vis spectra of the posolyte solution of $(TEA)_2$**Co**$_{mnt}$ (red to yellow with time), diluted by a factor of 20 to 50 µM **Co**$_{mnt}^{n-}$ in 5 mM $TBAPF_6$ MeCN solution, after an initial charge cycle to 2.2 V. Also shown is the uncharged starting solution (grey), $(TEA)_2$**Co**$_{mnt}$ diluted by a factor of 20 to 50 µM in 5 mM $TBAPF_6$ MeCN solution.

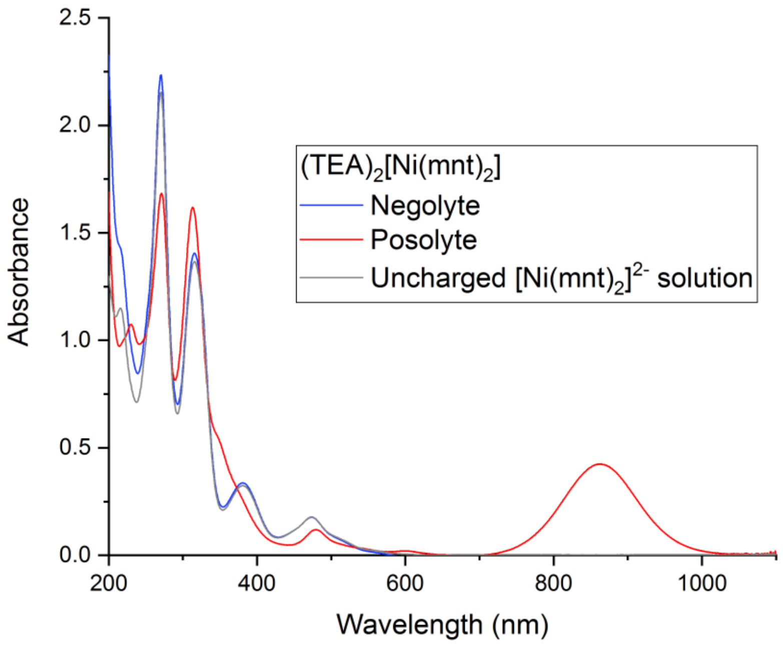

Figure S39. UV-vis spectra of the negolyte (blue) and posolyte (red) solutions of $(TEA)_2$**Ni**$_{mnt}$ diluted by a factor of 20 to 50 µM **Ni**$_{mnt}^{n-}$ in 5 mM $TBAPF_6$ MeCN solution, after an initial charge cycle to 2.4 V. Also shown is the uncharged starting solution of each half cell (grey), $(TEA)_2$**Ni**$_{mnt}$ diluted by a factor of 20 to 50 µM in 5 mM $TBAPF_6$ MeCN solution.

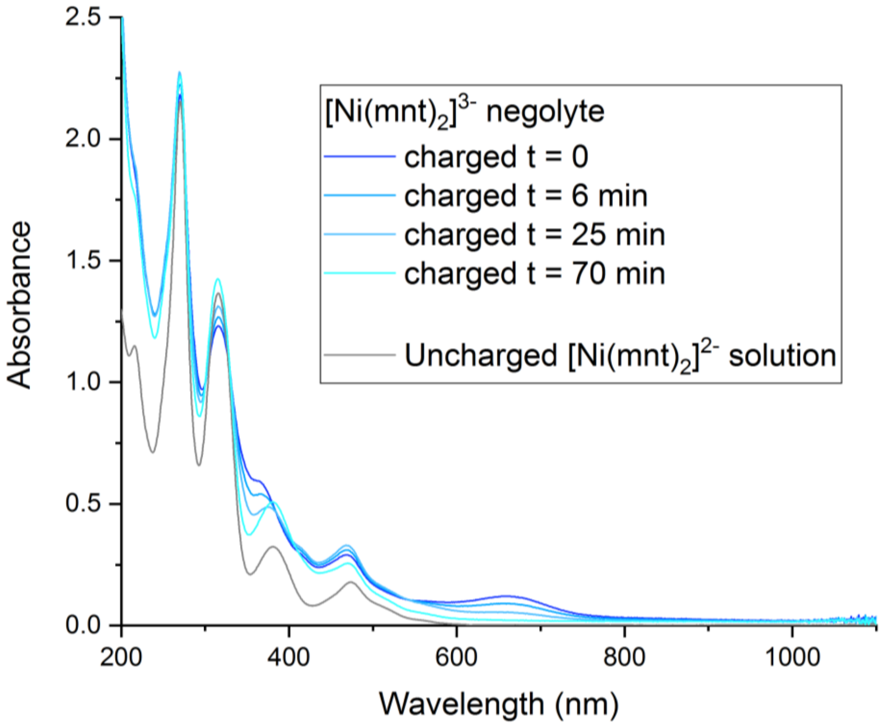

Figure S40. Time evolution of the UV-vis spectra of the negolyte solution of $(TEA)_2$**Ni**$_{mnt}$ (dark to light blue with time), diluted by a factor of 20 to 50 µM **Ni**$_{mnt}^{n-}$ in 5 mM $TBAPF_6$ MeCN solution, after an initial charge cycle to 2.4 V. Also shown is the uncharged starting solution (grey), $(TEA)_2$**Ni**$_{mnt}$ diluted by a factor of 20 to 50 µM in 5 mM $TBAPF_6$ MeCN solution.

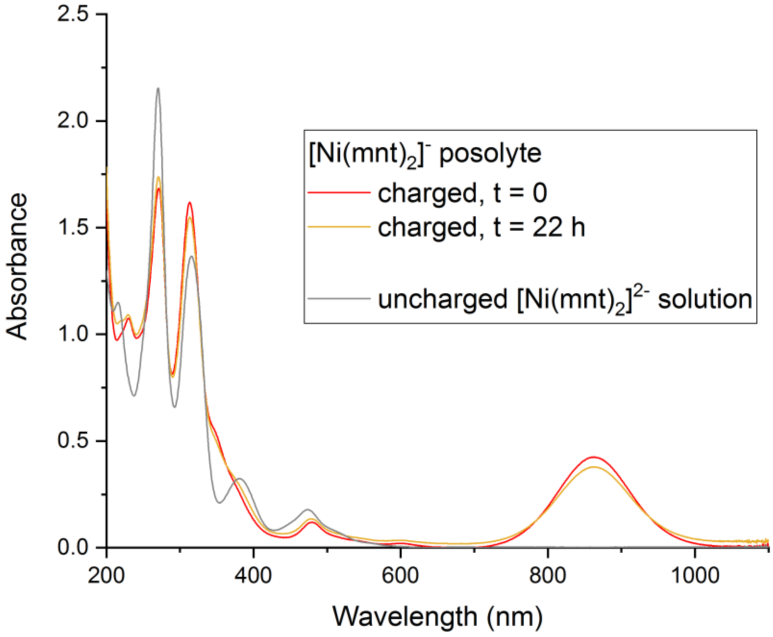

Figure S41. Time evolution of the UV-vis spectra of the posolyte solution of $(TEA)_2$**Ni**$_{mnt}$ (dark to light blue with time), diluted by a factor of 20 to 50 µM **Ni**$_{mnt}^{n-}$ in 5 mM $TBAPF_6$ MeCN solution, after an initial charge cycle to 2.4 V. Also shown is the uncharged starting solution (grey), $(TEA)_2$**Ni**$_{mnt}$ diluted by a factor of 20 to 50 µM in 5 mM $TBAPF_6$ MeCN solution.

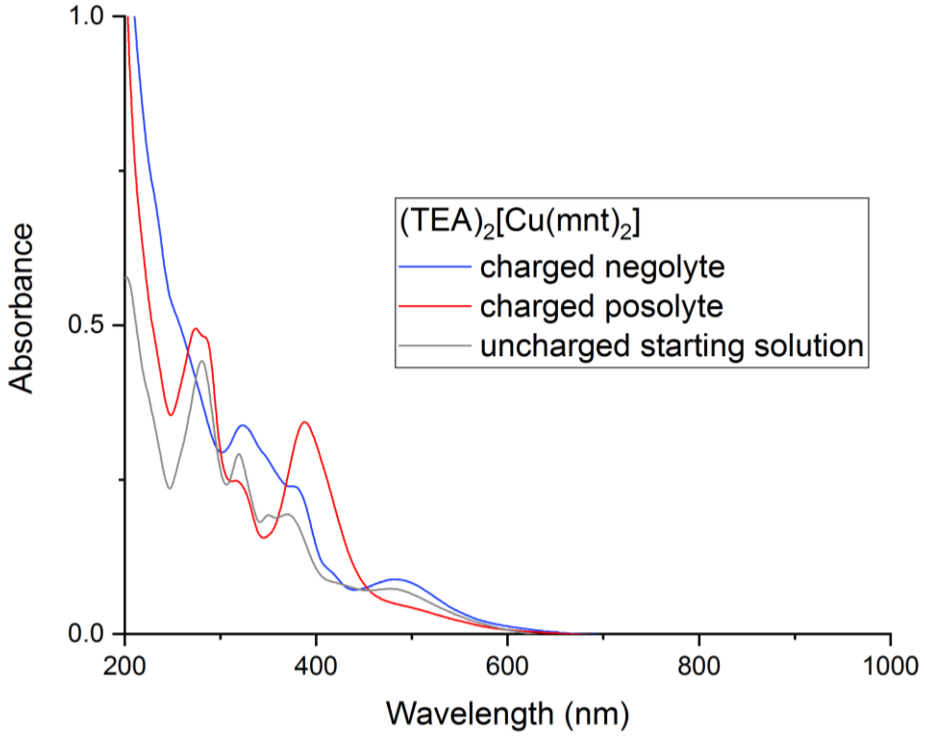

Figure S42. UV-vis spectra of the negolyte (blue) and posolyte (red) solutions of $(TEA)_2$**Cu**$_{mnt}$, diluted by a factor of 20 to 50 µM **Cu**$_{mnt}^{n-}$ in 5 mM $TBAPF_6$ MeCN solution, after an initial charge cycle to 1.6 V. Also shown is the uncharged starting solution of each half cell (grey), $(TEA)_2$**Cu**$_{mnt}$ diluted by a factor of 20 to 50 µM in 5 mM $TBAPF_6$ MeCN solution.

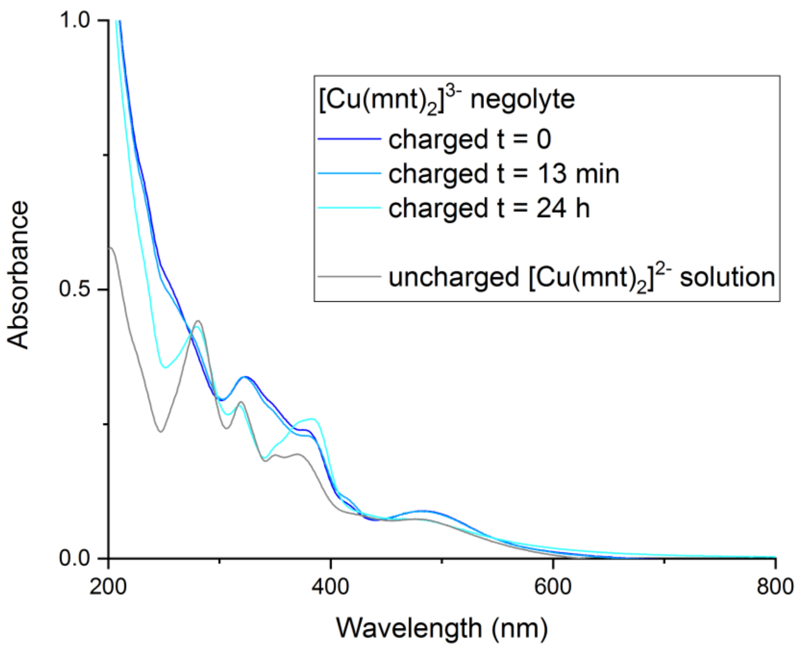

Figure S43. Time evolution of the UV-vis spectra of the negolyte solution of $(TEA)_2$**Cu**$_{mnt}$ (dark to light blue with time), diluted by a factor of 20 to 50 µM **Cu**$_{mnt}^{n-}$ in 5 mM $TBAPF_6$ MeCN solution, after an initial charge cycle to 1.6 V. Also shown is the uncharged starting solution (grey), $(TEA)_2$**Cu**$_{mnt}$ diluted by a factor of 20 to 50 µM in 5 mM $TBAPF_6$ MeCN solution.

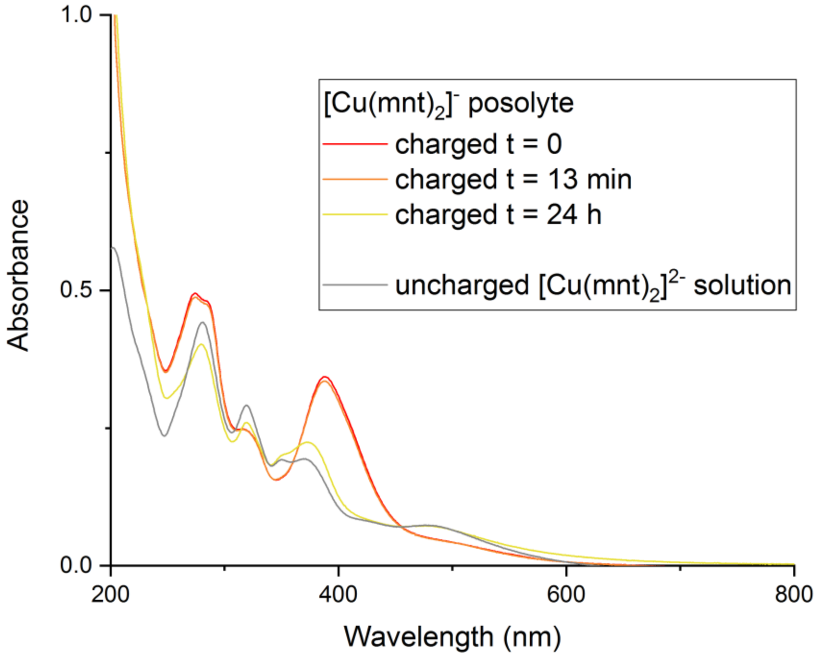

Figure S44. Time evolution of the UV-vis spectra of the posolyte solution of $(TEA)_2$**Cu**$_{mnt}$ (red to yellow with time), diluted by a factor of 20 to 50 µM **Cu**$_{mnt}^{n-}$ in 5 mM $TBAPF_6$ MeCN solution, after an initial charge cycle to 1.6 V. Also shown is the uncharged starting solution (grey), $(TEA)_2$**Cu**$_{mnt}$ diluted by a factor of 20 to 50 µM in 5 mM $TBAPF_6$ MeCN solution.

References

[1] A. Hoepping, R. Mengel, R. Mayer, *J. Prakt. Chem./Chem.-Ztg.* **1998**, *340*, 269-270.

[2] M. Glodde, S. Liu, P. R. Varanasi, *J. Photopolym. Sci. Technol.* **2010**, *23*, 173-184.

[3] P. J. Cappillino, H. D. Pratt, N. S. Hudak, N. C. Tomson, T. M. Anderson, M. R. Anstey, *Adv. Energy Mater.* **2014**, *4*, 1300566.

[4] A. Davison, N. Edelstein, R. H. Holm, A. H. Maki, *J. Am. Chem. Soc.* **1964**, *86*, 2799-2805.

[5] J. F. Weiher, L. R. Melby, R. E. Benson, *J. Am. Chem. Soc.* **1964**, *86*, 4329-4333.

[6] A. Davison, R. H. Holm, R. E. Benson, W. Mahler, in *Inorganic Syntheses, Vol. 10* (Ed.: E. L. Muetterties), McGraw-Hill, Inc., **1967**, pp. 8-26.

[7] J. D. Milshtein, K. M. Tenny, J. L. Barton, J. Drake, R. M. Darling, F. R. Brushett, *J. Electrochem. Soc.* **2017**, *164*, E3265-E3275.

[8] J. D. Milshtein, J. L. Barton, R. M. Darling, F. R. Brushett, *J. Power Sources* **2016**, *327*, 151-159.
